# Metascan: METabolic Analysis, SCreening and ANnotation of Metagenomes

**DOI:** 10.3389/fbinf.2022.861505

**Published:** 2022-06-22

**Authors:** Geert Cremers, Mike S. M. Jetten, Huub J. M. Op den Camp, Sebastian Lücker

**Affiliations:** Department of Microbiology, RIBES, Radboud University, Nijmegen, Netherlands

**Keywords:** metagenomics, metabolism, annotation, microbiology, ecology

## Abstract

Large scale next generation metagenomic sequencing of complex environmental samples paves the way for detailed analysis of nutrient cycles in ecosystems. For such an analysis, large scale unequivocal annotation is a prerequisite, which however is increasingly hampered by growing databases and analysis time. Hereto, we created a hidden Markov model (HMM) database by clustering proteins according to their KEGG indexing. HMM profiles for key genes of specific metabolic pathways and nutrient cycles were organized in subsets to be able to analyze each important elemental cycle separately. An important motivation behind the clustered database was to enable a high degree of resolution for annotation, while decreasing database size and analysis time. Here, we present Metascan, a new tool that can fully annotate and analyze deeply sequenced samples with an average analysis time of 11 min per genome for a publicly available dataset containing 2,537 genomes, and 1.1 min per genome for nutrient cycle analysis of the same sample. Metascan easily detected general proteins like cytochromes and ferredoxins, and additional *pmoCAB* operons were identified that were overlooked in previous analyses. For a mock community, the BEACON (F1) score was 0.72–0.93 compared to the information in NCBI GenBank. In combination with the accompanying database, Metascan provides a fast and useful annotation and analysis tool, as demonstrated by our proof-of-principle analysis of a complex mock community metagenome.

## Introduction

Alongside the advances in DNA sequencing, genome annotation has come a long way. Metagenomic sequence data are becoming available at increasing rates, making accurate and fast (automated) analysis tools even more important. Through the advancements of sequencing technologies, a single isolated bacterium prior to sequencing is not a requirement anymore, leading to an increase in the sequencing of metagenomes. This, in turn, leads to new challenges in annotation. It is common for metagenomes to be binned prior to annotation into metagenome-assembled genomes (MAGs). Especially when samples are (ultra-)deep sequenced, the number of MAGs per sample can reach thousands of near-complete genomes (Anantharaman et al., 2016). Not only do all these MAGs need to be annotated individually, which is time and effort consuming, there is also the greater ecological question of how the metabolic processes in the original sample relate to one another.

Additionally, there is the problem of protein ortho- and paralogs, which is especially prevalent when metagenomes lack enough sequencing depth for binning. Genes in a single genome are often distinct enough for a meaningful annotation, especially since for small genomes direct comparison like BLAST analysis (Altschul et al., 1990) to a database is still feasible. However, using BLAST on complex metagenomes is too computationally intense and time-consuming, and this will increase in the future, as databases keep growing every day (Evanko, 2009). Therefore, a faster, indirect comparison is preferred like the use of hidden Markov models (HMM), where annotation is based on matching amino acid patterns rather than whole gene or protein sequences. However, these patterns are very similar for ortho- and paralogs that have similar evolutionary origins (Jensen, 2001), which makes HMM databases with high resolution a necessity to achieve optimal annotations. Automated annotation is often dividing the process in single, specific functions like gene-calling, ribosomal RNA gene identification, and gene annotation. The results of the single analyses are subsequently combined in so called wrapper-scripts. For bacterial genomes, Prokka (Seemann, 2014) is probably the most well-known and fastest pipeline used at the moment. In recent years, scripts have been published that are able to annotate multiple genomes simultaneously, often by using well established databases like PFAM (Mistry et al., 2021), KOFAM (Aramaki et al., 2020), and TIGRFAM (Haft et al., 2013). Examples of these are METABOLIC (Zhou et al., 2019), DRAM (Shaffer et al., 2020), and eggNOG-mapper v2 (here-after eggNOG) (Cantalapiedra et al., 2021).

Here, we report on the construction of a new database by first clustering proteins for each KO number of the KEGG pathway database (Kanehisa and Goto, 2000) involved in central metabolic functions and subsequently building HMM profiles for each cluster. Key genes of major metabolic pathways were organized in pathway-specific individual databases (subsets), based on the grouping of Anantharaman et al. (2016). These databases together with a modified version of Prokka were then used for a gene-centric annotation and analysis of a mock community and previously published (meta-)genomes, either for all MAGs separately, or the unbinned assembly.

## Materials and Methods

### Database Creation

For the creation of the database, all KO numbers from the KEGG database that are part of metabolic pathways (“09100 Metabolism”; https://www.genome.jp/brite/ko00001) were collected and linked to Uniprot entries through LINKDB (https://www.genome.jp/linkdb)*.* For KO numbers with more than three entries, the entries were downloaded from the TrEMBL UniProt database (release 2018–09) (Bateman, 2019) and converted into multi-FASTA files. The sequences were filtered on length by calculating the average sequence length for each KO number, after which sequences longer than 150% and shorter than 60% of the average sequence length were discarded. If a set consisted of less than three sequences after length filtering, the unfiltered set was used.

For sequence de-replication, sets containing more than three entries were clustered (nearest neighbor) using Linclust from the MMSeq2.0 package (settings: -v 0 --kmer-per-seq 160 --min-seq-id 0.5 --similarity-type 1 --sub-mat blosum80. out --cluster-mode 2 --cov-mode 0 -c 0.7) (Steinegger and Söding, 2018). For each KO-number, clusters with less than three sequences were combined into 1 cluster. If less than three unique sequences were left after de-replication, the entire KO number was discarded. Subsequently, all resulting sequences for each KO number cluster were aligned individually using mafft v7 (settings: --quiet --anysymbol) (Katoh and Standley, 2013) and HMM profiles were created using hmmbuild (default settings) (Eddy, 2011).

Subsets with key genes for each metabolic pathway were created automatically based on KEGG classification (“09102 Energy metabolism”) and manually curated where possible (Supplementary Data S2) based on the functional classification described in Anantharaman et al. (2016). HMM profiles for hydrogenases were created by downloading FASTA files for each hydrogenase group from the HydDB website (Søndergaard et al., 2016) followed by HMM profile creation as described above.

### Metascan

Metascan expects a folder containing one or more DNA sequence files in FASTA format, where each file represents either an unbinned assembly (metagenome contigs) or a single MAG. When analyzing a complete unbinned metagenome, Metascan will generate an overview of all metabolic pathways and nutrient cycles. If the metagenome was binned, providing all MAGs allows annotation of each MAG. When using MAGs as input, the unbinned sequences (and, if applicable, small contigs discarded after size-filtering) are expected to be included as one or multiple separate bins, since a full gene-centric analysis of a metagenome is also dependent on the unbinned fraction of the microbial population that may exist in the sample.

### Procedure

**FIGURE 1:**
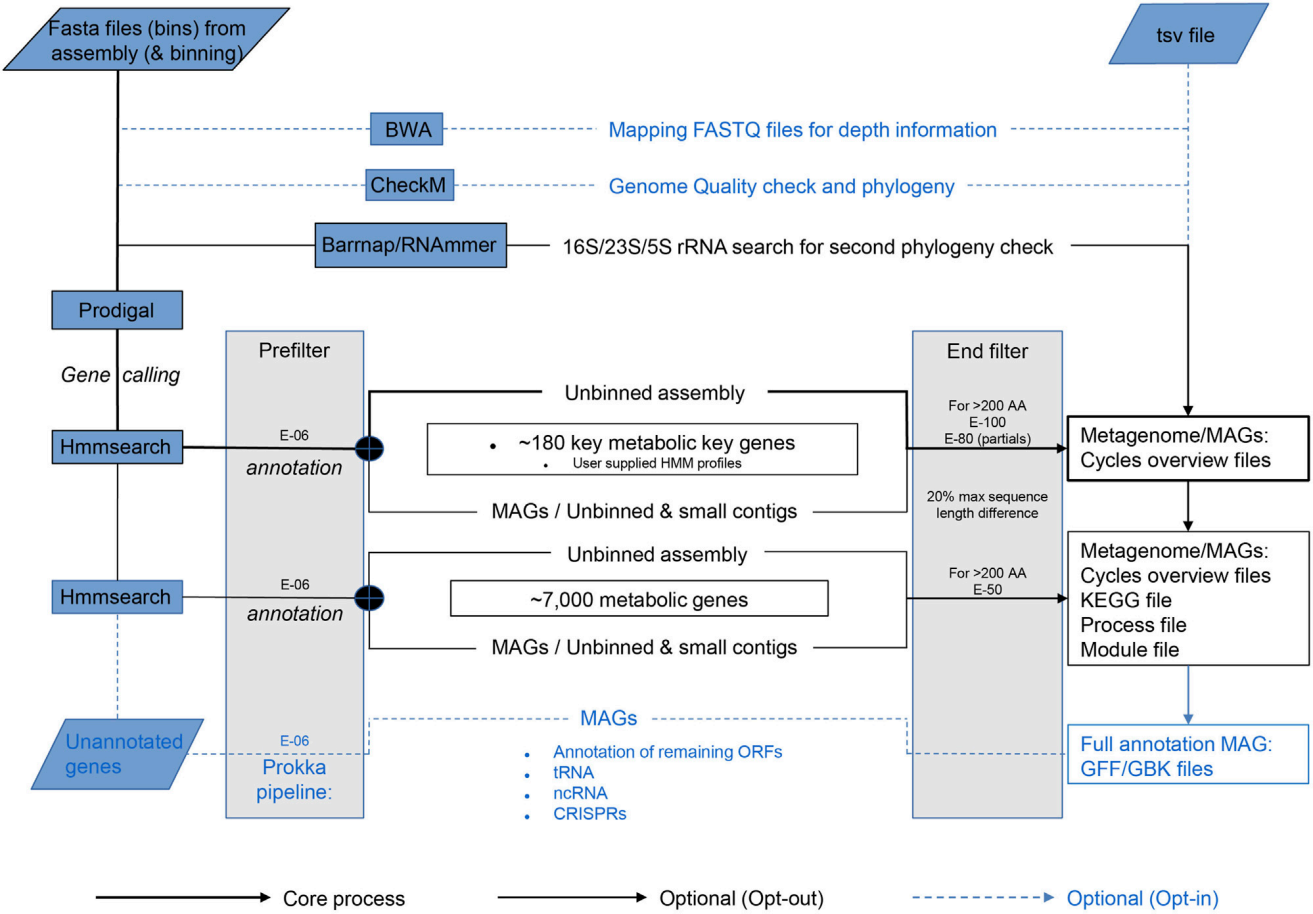
Schematic overview of the Metascan pipeline. The bold black lines represent the core analyses of the program. The thin black lines show the default Metascan pipeline when no options are opted out. The blue dotted lines indicate the options that can be invoked through the command line for full annotations.

The core process starts with gene calling by Prodigal (Hyatt et al., 2010) (Figure 1). Per default, Metascan runs a few additional analyses that can be excluded if a fast overview of the nutrient cycles present in the ecosystem is desired. Before annotation, a ribosomal RNA gene search is performed by either Barnnap (https://github.com/tseemann/barrnap) or RNAmmer (Lagesen et al., 2007). The recovered rRNA gene sequences are compared against a local NCBI nr database using BLASTN (Sayers et al., 2019). Subsequent gene annotation is performed using hmmsearch (Eddy, 2011) against each of the seven subsets of the key genes representing important nutrient cycles [Nitrogen, Methane, Carbon fixation, Hydrogenases, C1 (methylotrophy) molecules, Sulfur, and Oxidative phosphorylation; Table 2] and one miscellaneous subset of metal cycling. After annotation of the key genes, the remaining open reading frames (ORFs) are annotated using the HMM profiles of the remaining metabolic genes. If the metagenome was previously binned and abundance was estimated, this data can be entered in a separate TSV file.

For a full annotation of MAGs, the option—prokka is available. This Prokka legacy option provides tRNA search Aragorn (Laslett and Canback, 2004), ncRNA scan Infernal (Nawrocki and Eddy, 2013), and CRISPR scan Minced (Bland et al., 2007), exactly as Prokka (Seemann, 2014) would. It also annotates the remaining unidentified ORFs using BLASTP and the Prokka internal database. These options are also available individually.

### Bin Size

Metascan uses bin size in two different ways. First, for optimized gene calling, Prodigal has a single genome or metagenome mode. Thus, Metascan must determine whether the bin can be considered as single trustworthy MAG. Since the largest known bacterial genome is currently a little under 14.8 Mbp (Han et al., 2013), the maximum size for a bin to be considered a single prokaryotic genome is 15 Mbp. Anything larger is regarded as metagenomic by Metascan. Furthermore, for Prodigal the lower limit of the bin size is set at 0.5 Mbp, as this is the minimum Prodigal requires for gene-calling in single mode. Thus, bins smaller than 0.5 Mbp and larger than 15 Mbp are processed in meta mode. Prodigal in Metascan is also set to predict partial genes at the ends of contigs, as these are expected to be abundant in a metagenome. Secondly, the maximum bin size is also used to limit runtime by preventing time-consuming analyses like tRNA, ncRNA, CRISPR, and BLAST searches against small and unbinned contigs, as well as the unbinned metagenome.

### E-values

Like bin size, the e-value settings are important for the final outcome. Three different e-values are implemented in the Metascan workflow (Figure 1). The first and lowest e-value serves as a prefilter for HMM results to reduce the amount of working data. Here, E-06 is the highest score corresponding to the lowest protein identity allowed by Metascan, and this e-value is also used by all other first-level analyses. Next, Metascan differentiates between the application of the full metabolic dataset or the key gene set only. If only the smaller key genes databases are applied, the stringency is set to a more stringent setting of E-100 to exclude large numbers of false positives. When including the larger metabolic database, the stringency is lowered to E-50 because the risk for false positives is reduced by the probable presence of genes with higher similarity in the database, and a lower e-value here is useful to avoid false negatives. Simultaneously, the program applies a filter on size difference of ≥20% (by default) between target (as calculated by Hmmbuild during HMM construction) and query sequence to remove hits that clearly differ in size, but which contain similar sequence motifs. After all databases are queried, the hit with the highest bit-score is selected for each ORF.

For small proteins (<200 amino acids), the only e-value considered is the prefilter. Short sequences are not long enough to build up enough bit-score, resulting in large e-values even when the similarity is high. Since this will also include incomplete partial genes missing a start or stop codon, the hits are selected on size difference between target and query of maximum 30%. If desired, all target e-values can be set manually. As a final option, the program also accepts user generated HMM profiles, both as single input or in combination with the existing databases.

### Output

For each analysis, an overview file is produced that contains the number of hits for each gene of each nutrient cycle and the number of bins/MAGs harboring these genes, alongside their relative abundance (Supplementary File S1.1). For both genes and organisms, the absolute and relative coverage is provided, if applicable. A Krona (Ondov et al., 2011) HTML file is produced for visual reference. A TSV file is generated containing all protein hits for easy retrieval of proteins of interest. Finally, two more TSV files are created, containing the genes for each process and metabolic module, as used by KEGG mapper (Kanehisa and Sato, 2020) on the genome.jp website. The process file can be used to manually create a cycle diagram using the provided blank cycle diagram (Supplementary File S1.2).

For each bin, an overview file is produced with the number of hits for each gene and phylogenetic information if applicable. A file containing hits of all the detected KEGG numbers is created, which can be entered into KEGG mapper for further analysis. Two files containing hits against the database and statistics like bitscore and output from hmmsearch are retained as well. One file contains all possible hits, the other file is an overview of all the highest scoring hits. Furthermore a few additional files are created, including a file containing all ribosomal RNA genes and a tab-separated file with annotated genes for easy retrieval. Finally, a few FASTA, statistical, log and GenBank files are created, similar to standard Prokka output (Supplementary File S1.1).

### Validation

#### Mock Community

For eight different microorganisms, representing different metabolic traits, the genomes (Table 1) were downloaded and fully annotated using Metascan four ways: as separate genome bins or as a single simulated metagenome and using either only key-genes or the whole metabolic set (Table 2). The mock metagenome was simulated using CAMISIM (Fritz et al., 2019) on all eight genomes (default settings). Both the simulated metagenome and the eight genomes were also analyzed using METABOLIC (default settings), DRAM (default settings), Prokka and eggNOG (hmmer method and default settings).

**TABLE 1:** Genomes used in the mock community of this study.

Organism	Size (bp)	Topology	Accession number	Metabolism
*Methanosarcina acetivorans str. C2A*	5,751,492	Circular	AE010299	Methanogen
*Nitrosomonas eutropha C91*	2,781,824	Circular + plasmids	CP000450	Autotrophic ammonia-oxidizer
*Paracoccus denitrificans PD1222*	5,236,194	Circular + plasmids	CP000489	Denitrifier and methylotroph
*Escherichia coli str. K-12 substr. MG1655*	4,641,652	Circular	NC_000913	Heterotroph
*Candidatus* Methylomirabilis oxyfera	2,752,854	Circular	FP565575	Denitrifying methanotroph
*Nitrospira moscoviensis strain NSP M-1*	4,589,485	Circular	NZ_CP011801	Autotrophic nitrite-oxidizer
*Methylacidiphilum fumariolicum SolV*	2,476,671	Circular	NZ_LM997411	Nitrogen fixing methanotroph
*Candidatus* Kuenenia stuttgartiensis MBR1	4,406,153	Circular	NZ_LT934425	Anammox

**TABLE 2:** Overview of different analysis options, analysis times and properties per dataset. Time is the total analysis time. Pathways indicate whether the results are ordered by ecological pathways and processes in the output. Abundance shows the option to include depth values into the analysis and GBK indicates the state of the Genbank file that is created by the program.

Dataset		Metascan key genes	Metascan Full annotation	DRAM	eggNOG	METABOLIC	Prokka
8 genomes	Time	01 h 01	4 h 46	3 h 26	2 days 7 h 02	0 h 39	0 h 16
	Pathways	Yes	Yes	Individual	no	Individual	No
	Abundance	NA	NA	NA	NA	NA	NA
	GBK	full	full	No genes	No RNA	No genes	full
Simulated meta-genome	Time	1 h 08	2 h 54	1 h 06	2 days 09 h 18	0 h 50	0 h 10
	Pathways	yes	yes	yes	no	yes	No
	Abundance	NA	NA	NA	NA	NA	NA
	GBK	limited	limited	No genes	No RNA	No genes	full
2,537 genomes	Time	2 days 22 h 29	19 days 08 h 21	34 days 13 h 2	NP	3 days 11 h 01	NP
	Pathways	Yes	Yes	Individual		Individual	
	Abundance	Yes	Yes	No		no	
	GBK	full	full	No genes		No genes	
Unbinned meta-genome	Time	1 day 23 h 06	12 days 09 h 57	36 days 23 h 42^a^	Over 44 days^b^	3 days 17 h 28	NP
	Pathways	Yes	Yes	Yes		yes	
	Abundance	NA	NA	NA		NA	
	GBK	limited	limited	No genes		No genes	

NP, not performed; NA, not applicable.

a Program crashed and was manually resumed, missing one step in the process.

b The program run for over 44 days and was manually stopped.

To obtain an accurate list of key genes present in these genomes, each KO number in the metabolic core dataset was cross-referenced with the KO numbers present in KEGG for those organisms. For unclear or missing results, additional BLAST checks and manual searches in the NCBI GenBank files were performed. Since no golden standards exist for the used organisms, the GenBank files generated by Metascan, Prokka (default settings) and eggNOG were compared to the GenBank files from NCBI using BEACON (Kalkatawi et al., 2015) with an offset of 2%. METABOLIC did not create files that could be converted into Genbank files. DRAM created Genbank files, but no annotation was present. Therefore, both programs could not be included in the BEACON comparison.

The BEACON scores were found to be identical to F1 scores (Van Rijsbergen, 1977) and we consequently report the BEACON scores as F1 scores for the comparison of the different annotations (Supplementary Data S3).

#### Metagenome Analysis

2537 MAGs and the accompanying coverage data from the study by Anantharaman et al. (2016) were downloaded from ggKbase (https://ggkbase.berkeley.edu/2500-curated-genomes/organisms/). The key gene as well as full metabolic analyses were performed on the binned and unbinned genomes (Table 2). Both the binned and unbinned datasets were furthermore analyzed using METABOLIC (default settings) and DRAM (default settings). EggNOG accepted only a single FASTA file, and thus only the unbinned dataset was analyzed. The results of the Metascan analyses and the original study were manually compared by analyzing the statistics for the various nutrient cycles.

#### Computing Platform

All analyses were performed using 12 cores except for DRAM (10) on a server with one 32 core Intel(R) Xeon(R) CPU E5-2650 v2 @ 2.60 GHz and 227 G RAM.

#### Code and Data Availability Statement

Metascan can be obtained from https://github.com/gcremers/metascan, the required databases from Zenodo.org (https://doi.org/10.5281/zenodo.6365663).

## Results

### Database Creation

For the creation of the HMM database, 7,788 unique KO numbers associated with metabolic pathways were identified from file ko00000. keg (7 May 2018; renamed in KEGG to ko00001. keg in recent versions). When connecting these to proteins deposited in UniProt, 876 KO numbers had less than 3 UniProt entries available and were therefore excluded. Sequences from the remaining 6,912 KO numbers were downloaded from the UniProtKB/TrEMBL database, converted to FASTA format, and subjected to dereplication and length filtering (60%–150% of the mean length for each set). After dereplication, 46 sequence sets were discarded because a limited amount (<3) of unique sequences was left for alignment. Five unfiltered sets were retained as the length filtering step would have dropped the available sequences below three. In total, this left a final of 6,866 KO numbers available for alignment and HMM building.

After manually adding missing entries, subsets for each nutrient cycle were manually created (Table 3). For each key gene in a nutrient cycle, entries were manually checked and completed for lesser studied genes like hydrazine synthase. Finally, 38 profiles were calculated for hydrogenases by aligning sequences taken from HydDB (Søndergaard et al., 2016) for each (sub-)category.

**TABLE 3:** Number of genes per subset (cycle) and the number of corresponding HMM profiles.

#KO	Cycles	#HMM profiles
**38**	Hydrogenases	38
**25**	C1 molecules	319
**34**	Carbon fixation	643
**12**	Methane	32
**14**	Miscellaneous	213
**38**	Nitrogen	557
**14**	Oxygen	556
**40**	Sulfur	650
**6,739**	Non-key genes	114,157
**6,916**	Total	**117,127**

### Mock Community

We used DRAM, METABOLIC, eggNOG, and Prokka to analyze the original eight genomes and the CAMISIM simulated metagenome. We also used Metascan to analyze the eight genomes of the mock community using four different input and analysis settings (Table 2). Analysis times ranged from 16 min for all eight genomes (Prokka) to 2 days and 7 h for eggNOG; for the simulated metagenome this was from 10 min (Prokka) versus 1 day and 10 h for eggNOG. Metascan and Prokka both provided full GenBank files for further analysis, whereas eggNOG provided a GenBank files without RNAs. DRAM and METABOLIC did not include the annotation within the GFF file, which meant a meaningful GenBank file could not be constructed.

### Runtimes

When testing the mock community, we first needed to identify all genes belonging to the different nutrient cycle within the NCBI entries for each microorganism. This proved not to be straightforward, since in GenBank the annotations are not stored with these cycles in mind. We thus created the individual nutrient cycling profiles of the reference organisms by manually mining KO numbers from their annotations in KEGG and GenBank for metabolic key genes and compared these to the Metascan output. For a complete annotation of all eight genome bins including all ∼7,000 metabolic genes, the analysis took 4 h and 46 min, with an average of 35.6 min per genome bin. On the same system, it took 2 h and 54 min for the simulated metagenome, with the exclusion of several steps (tRNA, ncRNA, CRISPR detection, and BLASTP) in the process due to bin size. The key genes only analyses took 68 min for the simulated metagenome and 1 h and 1 min for the binned genomes.

### Gene-Centric Annotation

The manual key genes mining of the mock community against NCBI and KEGG yielded a total of 447 key genes for all eight genomes, with the Nitrogen cycle being the most abundant (117 genes) and enzymes involved in hydrogen metabolism the least (nine genes; Table 4).

**TABLE 4:** Number of genes retrieved from the GenBank files of the mock community and four different Metascan analyses, ordered by cycle. Percentages state the percentage relative to the total number of genes recovered from the GenBank files.

	Number of genes				
Nutrient cycle	GBK	Unbinned, key genes	Binned, key genes	Unbinned, full	Binned, full
Sulfur	65	70	77	67	71
Hydrogen	15	19	22	10	*12*
Methane	25	24	24	25	25
Nitrogen	117	108	117	114	127
Oxidative phosphorylation	53	55	60	54	59
C1	68	95	108	72	79
Carbon fixation	85	123	154	87	118
Miscellaneous	19	26	31	18	18
**All**	**447**	**520 (116.3%)**	**593 (132.7%)**	**447 (100.0%)**	**509 (113.9%)**

GBK: GenBank file from NCBI, Key genes: Analysis using only the key genes as reference. Full: Analysis using all metabolic genes as reference. Unbinned: simulated metagenome generated by CAMISIM., Binned: separate genomes from NCBI.

Overall, the total amount of key genes recovered from the mock community by Metascan varied from 133% (binned and key genes only) to 100% (simulated and all metabolic genes) compared to the GenBank annotations. Among the cycles, Hydrogen (67%–147%), C1 (methylotrophy; 106%–159%), Carbon fixation (102%–181%), and Miscellaneous (95%–163%) have the largest variability, whereas Sulfur (103%–119%), Methane (96%–100%), Nitrogen (92%–109%), and Oxidative phosphorylation (102%–113%) showed better congruency with the GenBank annotation. As could be expected, the analyses that used all metabolic genes from the KEGG dataset are more comparable to the GenBank annotations than the analyses using only key genes. Binning the mock metagenome into genome bins did not influence these results much.

When looking into the data in more detail (Supplementary Data S4), it became apparent that the majority of differences was caused by a few specific types of proteins, mainly ferredoxins, and cytochromes. *Cbb*_*3*_-type cytochrome *c* oxidase subunit III (K00406) was found 5 and 14 times by Metascan in the simulated metagenome full metabolic and binned key genes-only analyses, respectively, vs. three times in the GenBank annotations. A similar pattern was observed for the cytochrome *b*_556_-containing formate dehydrogenase subunit gamma (FdoI, K00127; 17 and 6 vs. 5), the Fe-S subunits of anaerobic carbon-monoxide dehydrogenase (CooF, K00196; 30 and 11 vs. 2) and arsenate oxidase (AoxA, K08355; 9 and 0 vs. 0). Another example is the Fe-S-containing beta subunit of formate dehydrogenase (FdoH and K00124), where both binned (19) and simulated metagenome key genes-only (15) Metascan analyses yielded a surplus of positive hits. However, BLASTP analysis of these proteins against the NCBI database identified 13 of them as NADH-quinone oxidoreductase subunit NuoF. Manual inspection of the input data (K00124) used to generate the FdoH HMM profiles (Supplementary File S1.3) showed that several entries in these protein clusters are labeled as NuoF, indicating either misannotated entries or unspecificity within this database entry.

Another group of gene annotations that deviated from the GenBank entries entailed group 4 Ni-Fe hydrogenases. Here, in the key genes-only annotation Metascan found seven proteins in addition to those predicted in NCBI. However, all seven proteins were apparently corresponding to NuoC or NuoD subunits of NADH dehydrogenase complexes and not true hydrogenases, as they also were lacking the catalytic Ni-binding motif, despite e-values of 0.0 to 9E-161 in the HydDB database search.

### Genome-Centric Annotation of Metagenome-Assembled Genomes

Besides the broad metabolic overview that Metascan provides on the metagenome level, an additional useful feature is the possibility for parallel single genome annotations during the analysis, which allows for immediate downstream analysis of genomic potential for any given MAG. For comparison of single genome annotations, we used BEACON to compare the annotations produced by Prokka, Metascan, and eggNOG for each genome used in the mock community to the GenBank files from NCBI with an offset of 2% (Table 5, Supplementary Data S5). BEACON (F1) scores range from 0.90–0.91 for *E. coli* to 0.72–0.73 *M. acetivorans*. The results are very similar for all three methods for all organisms, except for the eggNOG annotation of *P. denitrificans* (0.47), which strongly deviated from Prokka and Metascan (0.87). When comparing Metascan to the different approaches, eggNOG, and Prokka F1 scores range from 0.99 to 1, except for *P. denitrificans* (eggNOG, 0.55). The similarity scores to the NCBI annotations again range from 0.72 (*M. acetivorans*) to 0.91 (*E. coli*). These results show that Metascan, eggNOG, and Prokka annotations are very similar to each other and that all three equally differ from the NCBI GenBank files.

**TABLE 5:** BEACON (F1) scores comparisons of the GenBank files created by Prokka, Metascan, eggNOG, and NCBI for all eight genomes.

	NCBI^a^			Metascan^b^		
Genbank	Metascan	eggNOG	Prokka	NCBI	eggNOG	Prokka
*E. coli*	0.91	0.90	0.91	0.91	0.99	1
*M. fumariolicum SolV*	0.84	0.83	0.84	0.84	0.99	0.99
*Candidatus* K. stuttgartiensis	0.80	0.79	0.80	0.80	0.99	1
*N. eutropha*	0.83	0.82	0.83	0.83	0.99	0.99
*Candidatus* M. oxyfera	0.81	0.80	0.81	0.81	0.99	1
*M. acetivorans*	0.72	0.73	0.72	0.72	0.99	0.99
*N. moscoviensis*	0.80	0.79	0.80	0.80	0.99	0.99
*P. denitrificans*	0.87	0.47	0.87	0.87	0.55	1

a F1 score compared to the Genbank files from NCBI.

b F1 scores compared to the Metascan annotation.

### In-Depth Comparison Metascan vs. NCBI

Compared to Metascan annotations, the number of genes with function annotations in NCBI GenBank was higher for all samples (Table 6). This was caused by the higher number of (conserved) hypothetical proteins in the Metascan/Prokka annotations, as these programs use a conservative annotation regime. Annotations containing words like “conserved” and “containing” are labeled hypothetical, as there is no definitive known function for these proteins. As a result, there are more hypothetical proteins in the Metascan annotations and thus a lower degree of genes with assigned apparent functions.

**TABLE 6:** Direct and detailed comparison of the GenBank files from NCBI and Metascan. The differences in the grey area are related to the NCBI reference.

Gene calls	M. a	N. e	P. d	E. c	cM. o	N. m	M. f	cK. s
Detected identical	2,960	2,193	4,324	3,988	2,294	3,400	1,875	3,089
Detected similar	472	75	196	97	106	213	73	177
Unique to NCBI	1,118	379	653	452	742	896	400	833
Unique to Metascan	1,514	490	656	330	361	933	348	825
ΔrRNA	−1	0	0	0	0	−1	0	−1
ΔtRNA	57	0	2	2	0	2	1	0
ΔncRNA	0	−3	−2	−72	0	−2	−2	−3
Δframeshift/Pseudo	0	−343	−213	−86	−2	−109	−151	−281
ΔFunctional genes	−1,409	−559	−998	−1,052	−1,438	−648	−379	−797
Total Reference	4,550	2,687	5,173	4,537	3,142	4,509	2,348	4,099
Total Metascan	4,946	2,758	5,176	4,415	2,757	4,546	2,296	4,091

M.a = *M. acetivorans*, N. e = *N. eutropha*, P.d = *P. denitrificans*, E. c = *E. coli*, cM.o = “*Candidatus* M. oxyera”, N.m = *N. moscoviensis*, M. f = *M. fumariolicum SolV*, cK.s = “*Candidatus* K. stuttgartiensis”. Δ+, Metascan annotated more genes; Δ−, metascan annotated less.

For two organisms there was a larger difference in the amount of ORFs called by GenBank compared to the other two methods. The first was *M. acetivorans*, for which 4550 ORFs were predicted by GenBank and 4,946 by Metascan, which is a difference of 8% (396 ORFs). However, visualizing the ORFs of *M. acetivorans* in Artemis (Carver et al., 2012) (Supplementary File S1.4) indicated the presence of amber stop-codons (TAG) within several genes in the NCBI GenBank annotation. The substitution of a TAG stop codon by a sense codon is a codon usage variation which has been described in some microorganisms and ciliates (Tourancheau et al., 1995). As a matter of fact, the usage of the unusual amino acid pyrrolysine has first been described in a paper by Heinemann et al. (2009). When re-analyzing the genome with Metascan using a translation table that does not use TAG as stop codon like table 25, a more intuitive layout of the ORFs appeared, as well as a gene count that is closer to the GenBank file (4,631). BLASTx analysis of a few of these ORFs against the NCBI *nr* database showed that they had full length hits against database entries, which had either amino acid X or O (pyrrolysine) at the position of the stop codon in the query sequence (Supplementary File S1.4).

Contrastingly, in the annotation of *M. oxyfera* Metascan predicted 2757 ORFs, which are 385 less than in the GenBank file (3,142; 13% difference). When comparing the two analyses through Artemis, it becomes apparent that the NCBI GenBank file contains more small proteins (<200 amino acids) than the Metascan GenBank file. The reason for this could be the threshold setting (1E-06) for small proteins to be considered a true protein within Metascan.

Noteworthy are the 57 tRNAs in *M. acetivorans* found by Metascan that were not present in the GenBank entry. This exemplifies that also GenBank files are far from perfect, as was discussed before (De Simone et al., 2020). However, Metascan had difficulties in identifying pseudo-genes (up to 343 genes in *Nitrosomonas eutropha*) and ncRNAs (up to 72 in *E. coli*).

### Metagenome Analysis

For a metagenome analysis, 2,537 genomes from a large-scale metagenomic study of aquifer sediments (Anantharaman et al., 2016) were downloaded from ggKbase (https://ggkbase-help.berkeley.edu) together with a pre-parsed file containing the average coverage depth for each bin. The per-genome key gene analysis for all 2,537 genomes in this dataset took almost three full days to complete, with an average of 1.7 min per genome. In the full analysis using all metabolic genes, it took the script about 19.5 days, corresponding to an average of 11 min per genome. The key gene analysis of the unbinned metagenome (i.e., the combined bins) was finished in just under 2 days, which would equate to 1.1 min per genome (Table 2).

Similar to the mock community analyses, the formate dehydrogenase iron-sulfur-containing beta (K00124; 369, 1018, 973, and 951 hits in the Full Annotation (FA), Binned Key gene (BK), Unbinned Key gene (UK), and Full Unbinned (FU) analyses, respectively) and gamma subunits (K00127; 126 FA, 1413 BK, 1381 UK, and 454 FU), and the anaerobic carbon-monoxide dehydrogenase iron-sulfur subunit CooF (K00196; 654 FA, 1862 BK, 833 UK, and 766 FU) showed clear differences in gene counts. Furthermore, malyl-CoA ligase frequencies were overestimated in the key gene analyses. BLAST analysis of these indicated that the misannotated genes were actually succinyl-CoA ligases, a gene not included in the key gene set but present in the large metabolic set.

### Metascan vs. Reference

A direct comparison between the analyses from Anantharaman et al. (2016) and Metascan is hampered by different choices made during analyses, like which genes to include in the key gene set and how to define the nutrient cycles. However, a few things became apparent (Table 7; Supplementary File S1.5). For instance, when focusing on methylotrophy Metascan identified 82 enzymes related to the pyrroloquinoline quinone (PQQ)-dependent methanol dehydrogenases (MDH) in the binned key gene analysis, which were not reported in the original analysis. After curating the retrieved set for (nearly) full length genes, a tree was constructed (Felsenstein, 1985; Saitou and Nei, 1987; Jones et al., 1992; Kumar et al., 2016), revealing that most of these proteins are PQQ-dependent alcohol dehydrogenases from largely uncharacterized lineages within this protein family (Supplementary File S1.6). Anantharaman et al. (2016) found one organism (Burkholderiales bacterium RIFCSPLOWO2_12_67_14) putatively involved in methane oxidation, based on the presence of the genes encoding the particulate methane monooxygenase (*pmoCAB*). In the key genes-only analysis, Metascan found five *pmoB,* and one *pmoC* gene hits that could also be confirmed using BLAST. In the full metabolic annotation, Metascan found additional six *pmoA* and five *pmoC* genes. In total, these genes were divided over four species from the order Burkholderiales. Thus, besides the earlier mentioned species, the dataset contained three previously unrecognized Burkholderiales bacteria encoding particulate methane monooxygenase. From those three, two MAGs contained two complete *pmoCAB* operons and one was predicted to only encode *pmoA* and *pmoC*. However, a BLAST search on the gene directly downstream revealed that *pmoCA* is followed by an unrecognized *pmoB* in this organism as well. Based on the coverage of the four species containing the *pmoCAB* genes, methanotrophy is found in ca. 0.6% of the entire sample and 0.16% of the total number of organisms, and methylotrophy constitutes 0.82% and 0.84%, respectively. Correspondingly, malyl-CoA lyase (*mcl*), a marker gene for the serine pathway in methanotrophy and methylotrophy, had a total abundance of 1.7% and was detected in 0.1% of all organisms. While these findings expand the number of putative methane oxidizers present, it still indicates that methane oxidation is of minor importance in this aquifer ecosystem.

**TABLE 7:** |Results from the Anantharaman et al. (2016) study and Metascan binned key gene analysis. Groundwater and sediment sample annotations were taken are from Anantharaman et al. (2016).

	Groundwater		Sediment		Metascan	
	N# org	%O-Depth^a^	N# org	%O-Depth^a^	N# org	%O-Depth^a^
Carbon Cycle						
Carbon fixation	186	12	186	30	1022	38
Methanogenesis	0	0	0	0	0	0
Methanotrophy	0	0	0	0	5	<1
Methylotrophy	NA	<1	NA	<1	51	3
Hydrogen oxidation	356	22	356	45	400	14
Sulfur Cycle						
Sulfate reduction	21	<1	21	2	165	9
Sulfite reduction	21	<1	21	<1	724	32
Thiosulfate oxidation	77	7	77	9	199	10
Thiosulfate reduction	53	2	53	6	361	17
sulfite oxidation	51	3	51	8	83	6
sulfide oxidation	208	17	208	29	371	18
sulfur oxidation	157	13	157	14	2	<1
sulfur reduction	223	16	223	23	194	12
Nitrogen cycle						
Nitrogen fixation	54	3	54	1	87	5
Anammox	11	2	11	1	22	<1
ammonia oxidation	0	0	0	0	14	<1
Nitrite oxidation	85	8	85	15	265^a^	14^a^
DNRA	108	12	108	13	499^b^	22^b^
Denitrification						
Nitrate reduction	212	15	212	18	265^a^	14^a^
Nitrite reduction	150	23	150	21	159	7
Nitric oxide reduction	109	6	109	11	168	10
Nitrous oxide reduction	56	3	56	4	98	6

a %O-depth is the percentage of the organisms that can perform the process in absolute numbers (depth). For instance, 12% of every single bacteria/archaea can perform Carbon Fixation in Groundwater.

b The HMMs, in Metascan cannot distinguish between nitrate reductases and nitrite oxidoreductases.

c These are the numbers for the small subunit NirD. Large subunit NirB has N# 151 and 10% O-depth.

On the contrary, a process in the nitrogen cycle that appears to be over-predicted by Metascan is nitrate reduction to ammonium (both assimilatory and dissimilatory), which is mainly caused by large numbers of misannotated small subunits of the two main enzyme systems catalyzing nitrite reduction (*nirD* and *nrfH*). BLAST analyses showed that besides true *nirD* these genes encode diverse ferredoxins, Rieske 2Fe-2S proteins and dioxygenases.

### Metascan vs. METABOLIC and DRAM

The eggNOG analysis ran for over 44 days and was expected to run for over a year at 5 h per genome, therefore the analysis was not included into the metagenome analysis in this paper. METABOLIC and DRAM reported the results as lists of identified genes per genome and did not provide a combined overview of all analyzed genomes. However, for DRAM an overview could be created from the available data. The binned analysis took 31 days and 13 h, 12 days longer than Metascan. The unbinned analysis ran for 36 days and 23 h, after which it crashed due to memory issues during the creation of the GFF files. Nevertheless, the distillation of the annotation was possible with the annotation files that were produced so far. Strikingly, both DRAM analyses were nearly identical and can thus be reported as one (unbinned; Supplementary Data S6). In METABOLIC, the binned analysis ran for 3 days and 17 h, the unbinned analysis for 3 days and 11 h. As METABOLIC did not provide a full overview of the combined genomes only the unbinned dataset was used for comparison. Both METABOLIC and DRAM reported the results in KEGG numbers, which were used for making the comparisons.

Table 8 summarizes the annotation results, reporting the maximum number any single protein assigned to the respective process was detected, or the sum of all detected hydrogenases in the case of hydrogen metabolism. Overall, annotations are similar for all three methods, with a few exceptions. Most notably, DRAM did neither detect any methanol dehydrogenases, nor anaerobic ammonium oxidation (anammox). The high number for MDHs in the other methods is likely an overestimation, which was confirmed by BLAST analysis that indicated that the number of MDHs is more in line with the predicted methanotrophy genes (15–17 MDHs). DRAM also did not report several sulfur cycling processes. For thiosulfate oxidation, METABOLIC detects the largest number of genes (320 vs. ∼130 in Metascan and DRAM), but the lowest for thiosulfate reduction (133). Here, Metascan reports much higher numbers (684 and 1,372), followed by DRAM (234). However, these numbers especially for Metascan appear to be an overestimation, as they are only based on the detection of PhsA. In contrast, PhsB was detected 385 and 226 times by Metascan, and PhsC even only 0 and 25 times, However, none of these genes was included in METABOLIC or DRAM, hampering a comparison between methods.

**TABLE 8:** Results from Metascan (unbinned), Metabolic (unbinned), and DRAM (unbinned) analyses of the Anantharaman metagenome (2016).

	Metascan		METABOLIC	Dram
	key	full		
Carbon Cycle	#hits^a^	#hits^a^	#hits^a^	#hits^a^
Carbon fixation	1578	2776	1707	1686
Methanogenesis	0	0	0	0
Methanotrophy	6	8	5	6
Methylotrophy	99^b^	294^b^	66	0
Hydrogen formation^c^	557	545	471	
Hydrogen oxidation^d^	1370	2596	537	2008^e^
Sulfur Cycle				
Sulfate reduction	193	480^f^	127	124
Sulfite reduction	449	718	378	388
Thiosulfate oxidation	133	195	320	124
Thiosulfate reduction	684^g^	1372^g^	133	234
sulfite oxidation	152	317	45	
sulfide oxidation	491	877	587	
sulfur oxidation	2	3	2	
sulfur reduction	451	681	276	
Nitrogen cycle				
Nitrogen fixation	103	208	102	87
Anammox	53	90	60	0
Ammonia oxidation	6	8	6	6
Nitrite oxidation	294	537	162	198
DNRA	670	578	290	198
Denitrification				
Nitrate reduction	294	537	148	198
Nitrite reduction	168	358	201	195
Nitric oxide reduction	181	303	340	194
Nitrous oxide reduction	98	39	96	96

a Reporting the maximum number any single protein assigned to the respective process was detected.

b Combined XoxF, MxaF (both EC:1.1.2.7) and NDMA-dependent MDH (EC:1.1.99.37).

c Sum of all Fe-Fe hydrogenases.

d Sum of all Ni-Fe hydrogenases.

e Sum of all hydrogenases detected, as there is no distinction between Ni-Fe and Fe-Fe hydrogenases in DRAM.

f Inflated by AsrB, otherwise 338.

g Inflated by PhsA, otherwise 385 and 226, respectively, based on PhsB detection.

Finally, when comparing the two Metascan analyses with each other, it becomes apparent that the number of genes predicted in the full annotation is higher for almost all cycles, likely due to the higher e-value (E-50 vs. E-100) used in the full annotation.

## Discussion

### Database Construction

In this study, we present Metascan, a new tool for analysis of the metabolic potential of complex microbial communities. We developed this tool to enable researchers to obtain a fast but detailed and reliable overview of the main nutrient cycle reactions encoded by complex microbial communities in large environmental metagenomic datasets. This functionality currently is lacking in most annotation tools, which mainly focus on genome-centric analyses and rarely structure their output to give an overview of the biogeochemical nutrient cycles being catalyzed in the investigated environment. Moreover, the currently available databases used for similarity search-based annotations are too large to allow fast annotations of complete metagenomes, too unstructured to yield an overview of the nutrient cycles taking place, or, in the case of well-curated databases, also too small to offer the required resolution especially for environmental communities rich in uncultured and understudied microorganisms. We thus constructed a novel HMM-based database that not only allowed fast and accurate gene- or genome-centric annotation of complex metagenomes, but also categorized the identified protein-coding genes according to the relevant nutrient cycles.

### Metagenome Analysis

A direct comparison of different annotation tools is hampered by the choices made during the analysis and the reporting of the results. Genes with multiple subunits can be reported as present when all, some or just one subunit is present. Some processes are part of two pathways (e.g., carbon fixation in methanol metabolism), and some cycles are represented by multiple pathways (carbon fixation). Obviously, different choices have a direct impact on the results. For instance, some protein complexes with multiple subunits like the anaerobic sulfate reductase (ASR) consist of subunits rarely detected in the Metascan full annotation (AsrA and ArsC, both detected five times) and others that are likely overpredicted (AsrB, detected 480 times).

In general, Metascan reached a similar level of precision as the GenBank reference annotation, although it tended to overpredict certain functions. This was especially prevalent for annotations of cytochromes and ferredoxins, which are very common proteins in nature and participate in a wide range of metabolic reactions, not seldom with overlap and interchangeability in function. To this extent, while both cytochromes and ferredoxins contain conserved domains that can easily be recognized through bioinformatics, a large set of well annotated reference proteins is required to ensure their exact annotation. However, this level of resolution is not present in most databases, and many automatically annotated genomes contain mis-annotated genes or lack proper annotation altogether. These errors then are propagated through different databases, consequently leading to a reduced reliability of annotation also in conventional tools (Schnoes et al., 2009). An example of this is K00124 (FdoH) in the UniProtKB/TrEMBL database, where either 1) the UniProtKB/TrEMBL dataset is heavily misannotated and many true NuoF are wrongly categorized under K00124, 2) the protein entries identified by BLASTP in the GenBank database are wrongly annotated as NuoF and in fact are true FdoHs, which then would also indicate that in the GenBank files from the mock community NuoFs are underrepresented, or 3) these subunits belong to distinct protein complexes participating in different pathways but are too similar to be distinguished by HMM searches. While the last option seems plausible here, since NuoF and FdoH both are Fe-S proteins with a common evolutionary history (Oh and Bowien, 1998), this issue mainly appears to be caused by the propagation of misannotations in the public databases (Schnoes et al., 2009), as especially many formate dehydrogenase beta subunit genes appear to be deposited as NuoF in GenBank. Similarly, for the overestimated carbon monoxide dehydrogenase iron-sulfur subunit CooF (K00196), the raw data gathered from UniProtKB/TrEMBL contained mostly unnamed ferredoxins, which corresponds to a large part of the obtained false positives in our analyses.

Another factor hampering correct functional annotation can be overlapping functionality of enzymes. For instance, malyl- and succinyl-CoA ligases react with two structurally quite similar substrates, as both malate and succinate are small four-carbon dicarboxylic acids. Since both proteins furthermore catalyze the same type of reaction, they are structurally very similar with respect to their conserved regions, which is also reflected in the fact that succinyl-CoA ligase is able to use malate as alternative substrate (Nolte et al., 2014). Consequently, when using a small database as in our key genes-only analysis that contained only the malyl-CoA ligase, E-values for hits against succinyl-CoA ligases are small enough to be considered significant, leading to the observed overestimation of malyl-CoA ligases. For this particular case, this could largely be resolved by adding the succinyl-CoA ligase to the core gene set representing the citric acid. In general, this showcases the necessity of using databases with good resolution, but it also highlights the underlying intrinsic problem of annotating complex microbial communities, where the genes of novel microorganisms might be so distinct that an automatic differentiation between such similar functions is not possible.

Despite these imperfections in our HMM database, annotations with Metascan achieved a level of precision comparable to other annotation tools, but at a greatly reduced analysis time. In general, it is becoming increasingly challenging to obtain fast and reliable annotations due to the rapid growth of reference databases and the increasing size and sequencing depth of metagenomic samples to be analyzed. Thus, methods that reduce the reference dataset by clustering entries into subsets represented by HMM profiles are promising developments to overcome this hurdle, especially when considering that Metascan reached a high precision despite the drawbacks of the uncurated input database.

As indicated above, it became apparent during the development of this tool that we needed to construct a database that not only allows fast and accurate annotation of gene functions, but also categorizes the output according to the major nutrient cycles, which required a novel approach to build and structure this database.

### Further Considerations

Here, we opted for a proof-of-concept approach, based on clustering proteins deposited in the UniProtKB/TrEMBL database. This database is by no means perfect since many protein entries in TrEMBL are not correctly annotated or incomplete, and herein lies the major point of improvement of our HMM database. The ideal input dataset would be manually curated like for instance the UniProtKB/SwissProt database, which will vastly increase the correctness for annotation. However, such well-curated databases are not yet suitable as many KO numbers are represented by less than three entries, which is the minimal number of sequences needed to create a HMM profile. A solution to circumvent this limitation would be a top-down approach, starting from a well curated database and subsequently adding missing HMM profiles using entries from other, less-well curated data sources.

Another possibility to improve the reliability of annotation is by employing a more stringent trimming and clustering algorithm when building the HMM database. However, while creating a database with stricter clustering rules will increase correctness, this will be at the expense of a longer analysis time. Lastly, the proteins in our database were clustered based on similarity, but if clustering instead was achieved by means of phylogenetic trees, this would provide additional information not only about evolutionary descent, but also about the exact function of proteins belonging to large and diverse enzyme families. However, this comes with its own set of difficulties and is not a trivial matter.

In the future, similar HMM subsets as developed here for nutrient cycling metabolic pathways could be constructed for non-metabolic pathways for a more complete genomic annotation. This will however greatly increase the runtime of the script, which would mean the need for a heavier computational infrastructure. For virus detection, a database of viral genes could be constructed in a similar way as presented here. Furthermore, the same procedure might be applicable for cell loci-specific proteins (e.g., cell wall or S-layer spanning), as these often share stretches of conserved amino-acids. In combination with RNA-seq, our HMM-based annotation approach would not only detect metabolic potential, but also actual activity of the overall cycles.

All things considered, we feel that Metascan can be of great help in mapping the important nutrient cycling pathways in an ecosystem by reducing and simplifying the input databases without compromising accuracy.

## Data Availability

Publicly available datasets were analyzed in this study. This data can be found here: https://www.ncbi.nlm.nih.gov/bioproject/PRJNA288027 (NCBI BioProject PRJNA288027) or https://ggkbase.berkeley.edu/2500-curated-genomes/organisms (ggKbase).
