# Supplementary figures and images for: Metascan: METabolic Analysis, SCreening and ANnotation of Metagenomes
Source: Front Bioinform. 2022 Jun 22;2:861505. doi: 10.3389/fbinf.2022.861505 (PMC9580885; doi:10.3389/fbinf.2022.861505)

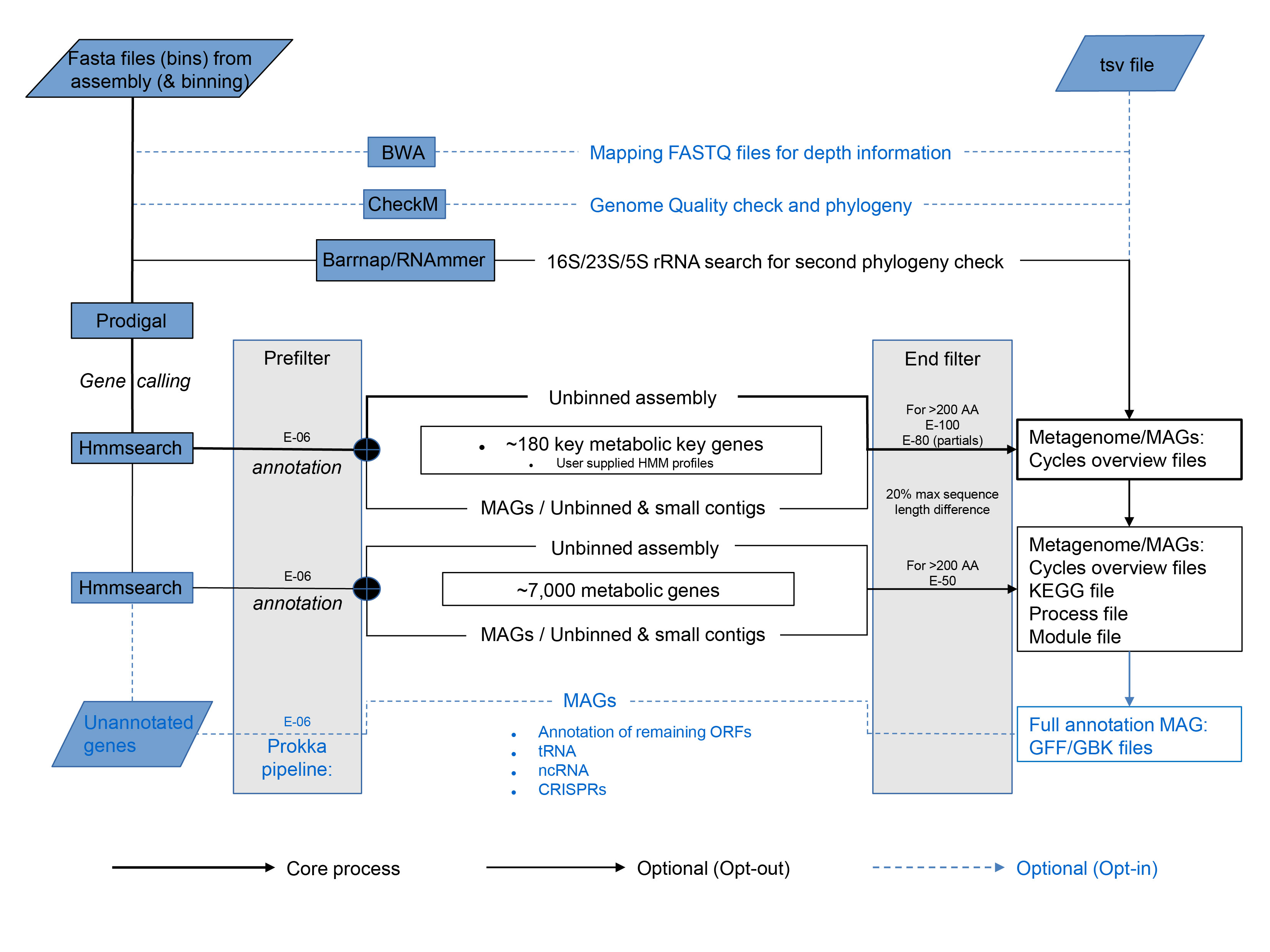

Supplement: Supplementary file 5 [file Figure1.JPEG]
