# Supplementary material for: Metascan: METabolic Analysis, SCreening and ANnotation of Metagenomes
Source: Front Bioinform. 2022 Jun 22;2:861505. doi: 10.3389/fbinf.2022.861505 (PMC9580885; doi:10.3389/fbinf.2022.861505)
Supplement: Supplementary file 7 [file DataSheet1.pdf]

Metascan: METabolic Analysis, SCreening and ANnotation of metagenomes

Geert Cremers[1], Mike S.M. Jetten[1], Huub J.M. Op den Camp[1], Sebastian Lücker[1]*

[1]Department of Microbiology, RIBES, Radboud University, Nijmegen, The Netherlands

Supplementary material S1

1 Output files of the Metascan program

2 Blank cycles diagram

3 Headers Fasta file NuoF/fdoH A0A126T7A4.16.faa

4 Gene calling of *Methanosarcina acetivorans str. C2A*

5 Cycles diagram Anantharaman metagenome

6 PQQ-MDH-Anantharaman phylogenetic tree

Supplementary file 1.1: Output files of the Metascan program

	Output file	Contents
Full metagenome	bin.id	Link between binname and Metascan ID
	depths.bins	List depth for each bin
	metagenome.tsv	List of every gene identified during the analysis
	krona.html	Krona file of cycles
	mod.tsv	List of identified genes in each modules
	proc.tsv	List of identified genes in each (sub) process
	ribosomal.ovw	List of every rRNA gene identified during the analysis
	total.tsv	TSV file of total statistics for each K-number
	total.ovw	File of total statistics for each K-number including KEGG database
Single genome or MAG	bin.all.faa	List of all amino-acids FASTAs recovered in this bin
	bin.all.ffn	List of all nucleic-acids FASTAs recovered in this bin
	bin.err	error file
	bin.f16	List of 5/16/23S genes
	bin.fall	List of all nucleic-acids FASTAs recovered in this bin including 5/16/23S genes
	bin.fna	File containing the FASTA file of the bin
	bin.fsa	File containing the FASTA file of the bin
	bin.gbk	GenBank file
	bin.gff	GFF file
	bin.hmm.faa	List of all amino-acids FASTAs recovered in this bin through HMM
	bin.hmm.ffn	List of all nucleic-acids FASTArecovered in this bin through HMM
	bin.kegg	List of KO numbers and genes, to be used with KEGG Mapper ()
	bin.log	Log file
	bin.ovw	File of total statistics for each K-number including rRNA
	bin.sqn	Sequin file
	bin.tbl	Table of CDS's
	bin.total.sort.tbl	Raw data HMM sorted
	bin.total.uniq.tbl	Raw data HMM sorted and de-replicated
	bin.tsv	File of K-number occurrences including KEGG database
	bin.txt	Basic statistics bin
	hydrogenases/	directory containing FASTA files of hydrogenases

Supplementary file 1.2: Blank cycles diagrams

A: Carbon

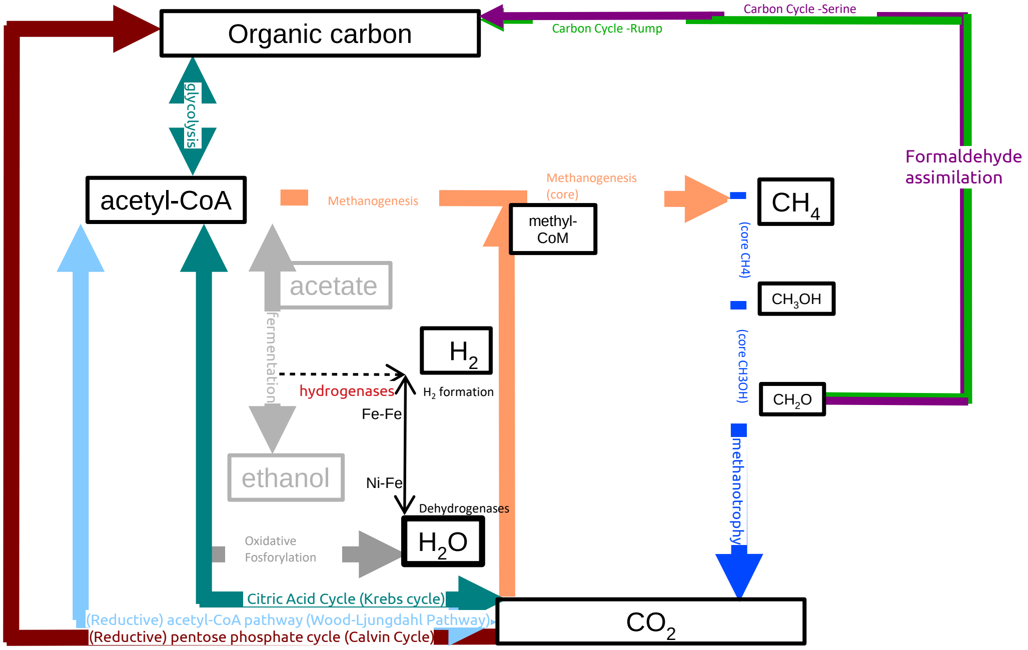

B: Sulfur

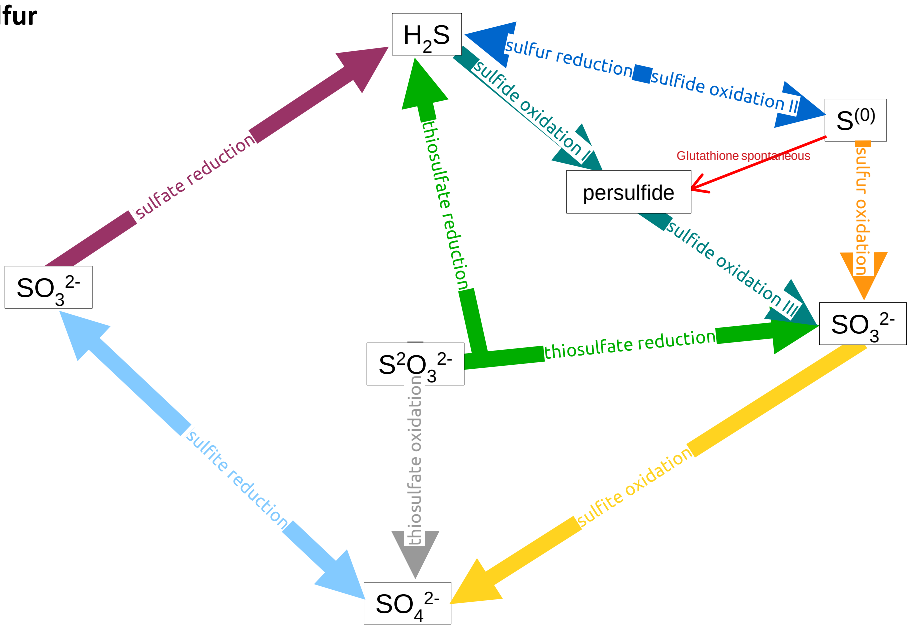

C: Nitrogen

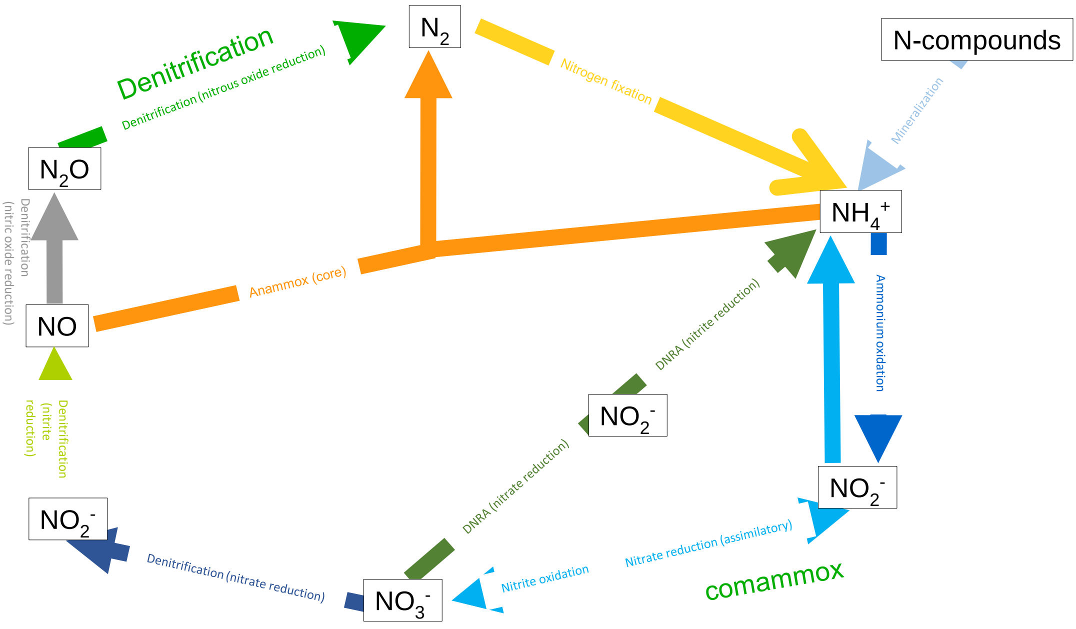

Supplementary file 1.3: Headers Fasta file NuoF/FdoH A0A126T7A4.16.faa

>tr|A0A126T7A4|A0A126T7A4_9GAMM Formate dehydrogenase {ECO:0000313|EMBL:AMK77956.1} OS=Methylomonas denitrificans GN=JT25_015965 {ECO:0000313|EMBL:AMK77956.1} PE=4 SV=1

>tr|A0A249LFW3|A0A249LFW3_9ACTN Formate dehydrogenase iron-sulfur subunit {ECO:0000313|EMBL:ASY27963.1} OS=Candidatus Planktophila limnetica GN=PHILAsVB114_04905 {ECO:0000313|EMBL:ASY27963.1} PE=4 SV=1

>tr|A0A0U3P4J4|A0A0U3P4J4_9RHOB Formate dehydrogenase {ECO:0000313|EMBL:ALV28582.1} OS=Pannonibacter phragmitetus GN=APZ00_17195 {ECO:0000313|EMBL:ALV28582.1} PE=4 SV=1

>tr|A0A172UC39|A0A172UC39_9GAMM Formate dehydrogenase {ECO:0000313|EMBL:ANE56704.1} OS=Methylomonas sp. DH-1 GN=AYM39_17000 {ECO:0000313|EMBL:ANE56704.1} PE=4 SV=1

>tr|D7DMN3|D7DMN3_METV0 NADH dehydrogenase (Quinone) {ECO:0000313|EMBL:ADI30810.1} OS=Methylotenera versatilis (strain 301) GN=M301_2448 {ECO:0000313|EMBL:ADI30810.1} PE=4 SV=1

>tr|A0A291HNA8|A0A291HNA8_9GAMM Formate dehydrogenase {ECO:0000313|EMBL:ATG73633.1} OS=Zobellella denitrificans GN=AN401_06995 {ECO:0000313|EMBL:ATG73633.1} PE=4 SV=1

>tr|A0A1Z4BXF1|A0A1Z4BXF1_9GAMM Formate dehydrogenase {ECO:0000313|EMBL:ASF45976.1} OS=Methylovulum psychrotolerans GN=CEK71_07715 {ECO:0000313|EMBL:ASF45976.1} PE=4 SV=1

>tr|A0A160N4D8|A0A160N4D8_9GAMM Formate dehydrogenase subunit beta {ECO:0000313|EMBL:AND70707.1} OS=Dyella thiooxydans GN=ATSB10_32530 {ECO:0000313|EMBL:AND70707.1} PE=4 SV=1

>tr|A0A0C5J9Z9|A0A0C5J9Z9_9RHOO Formate dehydrogenase {ECO:0000313|EMBL:AJP48770.1} OS=Rugosibacter aromaticivorans GN=PG1C_10640 {ECO:0000313|EMBL:AJP48770.1} PE=4 SV=1

>tr|V6F0D8|V6F0D8_9PROT NADH-quinone oxidoreductase subunit F, NuoF {ECO:0000313|EMBL:CDK98970.1} OS=Magnetospirillum gryphiswaldense MSR-1 v2 GN=nuoF {ECO:0000313|EMBL:CDK98970.1} PE=4 SV=1

>tr|A0A1A9EYX0|A0A1A9EYX0_9GAMM Formate dehydrogenase {ECO:0000313|EMBL:ANG62729.1} OS=Marinobacterium aestuarii GN=A8C75_09695 {ECO:0000313|EMBL:ANG62729.1} PE=4 SV=1

>tr|E1V907|E1V907_HALED Homolog to NADH-quinone oxidoreductase subunit NuoF {ECO:0000313|EMBL:CBV41782.1} OS=Halomonas elongata (strain ATCC 33173 / DSM 2581 / NBRC 15536 / NCIMB 2198 / 1H9) GN=HELO_1898 {ECO:0000313|EMBL:CBV41782.1} PE=4 SV=1

>tr|A3PNC6|A3PNC6_RHOS1 NADH dehydrogenase (Quinone) {ECO:0000313|EMBL:ABN77842.1} OS=Rhodobacter sphaeroides (strain ATCC 17029 / ATH 2.4.9) GN=Rsph17029_2740 {ECO:0000313|EMBL:ABN77842.1} PE=4 SV=1

>tr|A0A249KTV8|A0A249KTV8_9ACTN Formate dehydrogenase iron-sulfur subunit {ECO:0000313|EMBL:ASY20204.1} OS=Candidatus Planktophila vernalis GN=A7sIIA15_04955 {ECO:0000313|EMBL:ASY20204.1} PE=4 SV=1

>tr|E6SE61|E6SE61_INTC7 Formate dehydrogenase beta subunit {ECO:0000313|EMBL:ADU48709.1} OS=Intrasporangium calvum (strain ATCC 23552 / DSM 43043 / JCM 3097 / NBRC 12989 / 7 KIP) GN=Intca_2200 {ECO:0000313|EMBL:ADU48709.1} PE=4 SV=1

>tr|B9KP13|B9KP13_RHOSK NADH dehydrogenase {ECO:0000313|EMBL:ACM02335.1} OS=Rhodobacter sphaeroides (strain KD131 / KCTC 12085) GN=RSKD131_2475 {ECO:0000313|EMBL:ACM02335.1} PE=4 SV=1

>tr|A0A1U7D581|A0A1U7D581_9RHOB Formate dehydrogenase beta subunit {ECO:0000313|EMBL:APX23230.1} OS=Salipiger profundus GN=Ga0080559_TMP2434 {ECO:0000313|EMBL:APX23230.1} PE=4 SV=1

>tr|A0A1P8UVB3|A0A1P8UVB3_9RHOB Formate dehydrogenase beta subunit {ECO:0000313|EMBL:APZ53333.1} OS=Pelagibaca abyssi GN=Ga0080574_TMP2999 {ECO:0000313|EMBL:APZ53333.1} PE=4 SV=1

>tr|V9VQE5|V9VQE5_9RHOB Formate dehydrogenase subunit beta {ECO:0000313|EMBL:AHC99554.1} OS=Leisingera methylohalidivorans DSM 14336 GN=METH_01475 {ECO:0000313|EMBL:AHC99554.1} PE=4 SV=1

>tr|W6K700|W6K700_9PROT Putative formate dehydrogenase beta subunit FdsB {ECO:0000313|EMBL:CCQ72463.1} OS=Magnetospira sp. QH-2 GN=MGMAQ_0494 {ECO:0000313|EMBL:CCQ72463.1} PE=4 SV=1

>tr|F4CRF0|F4CRF0_PSEUX NADH dehydrogenase (Quinone) {ECO:0000313|EMBL:AEA25241.1} OS=Pseudonocardia dioxanivorans (strain ATCC 55486 / DSM 44775 / JCM 13855 / CB1190) GN=Psed_3042 {ECO:0000313|EMBL:AEA25241.1} PE=4 SV=1

>tr|C1DAU9|C1DAU9_LARHH NADH dehydrogenase {ECO:0000313|EMBL:ACO73280.1} OS=Laribacter hongkongensis (strain HLHK9) GN=LHK_00285 {ECO:0000313|EMBL:ACO73280.1} PE=4 SV=1

>tr|A0A127MDQ1|A0A127MDQ1_9SPHN Formate dehydrogenase {ECO:0000313|EMBL:AMO71268.1} OS=Sphingorhabdus sp. M41 GN=AZE99_04795 {ECO:0000313|EMBL:AMO71268.1} PE=4 SV=1

>tr|A0A2D3R976|A0A2D3R976_9SPHN Formate dehydrogenase {ECO:0000313|EMBL:ATW02235.1} OS=Sphingorhabdus sp. YGSMI21 GN=CHN51_00840 {ECO:0000313|EMBL:ATW02235.1} PE=4 SV=1

>tr|A0A1B3N489|A0A1B3N489_9SPHN NADH-ubiquinone oxidoreductase-F iron-sulfur binding region family protein {ECO:0000313|EMBL:AOG00191.1} OS=Blastomonas sp. RAC04 GN=BSY18_1368 {ECO:0000313|EMBL:AOG00191.1} PE=4 SV=1

>tr|G2IQU6|G2IQU6_9SPHN NAD-dependent formate dehydrogenase beta subunit {ECO:0000313|EMBL:BAK65968.1} OS=Sphingobium sp. SYK-6 GN=SLG_12930 {ECO:0000313|EMBL:BAK65968.1} PE=4 SV=1

>tr|A0A1X9YBH2|A0A1X9YBH2_9SPHN Formate dehydrogenase {ECO:0000313|EMBL:ARS26172.1} OS=Sphingomonas sp. KC8 GN=KC8_02555 {ECO:0000313|EMBL:ARS26172.1} PE=4 SV=1

>tr|A0A0C5L3R9|A0A0C5L3R9_9SPHN Formate dehydrogenase {ECO:0000313|EMBL:AJP72557.1} OS=Sphingomonas hengshuiensis GN=TS85_13405 {ECO:0000313|EMBL:AJP72557.1} PE=4 SV=1

>tr|G2J4E5|G2J4E5_PSEUL Formate dehydrogenase, beta subunit {ECO:0000313|EMBL:BAK76940.1} OS=Pseudogulbenkiania sp. (strain NH8B) GN=NH8B_2125 {ECO:0000313|EMBL:BAK76940.1} PE=4 SV=1

>tr|A1B5Z7|A1B5Z7_PARDP Formate dehydrogenase beta subunit {ECO:0000313|EMBL:ABL70941.1} OS=Paracoccus denitrificans (strain Pd 1222) GN=Pden_2857 {ECO:0000313|EMBL:ABL70941.1} PE=4 SV=1

>tr|A0A0N9NSL7|A0A0N9NSL7_9RHOB Formate dehydrogenase {ECO:0000313|EMBL:ALG91938.1} OS=Confluentimicrobium sp. EMB200-NS6 GN=TQ29_11090 {ECO:0000313|EMBL:ALG91938.1} PE=4 SV=1

>tr|A4WWH6|A4WWH6_RHOS5 NADH dehydrogenase (Quinone) {ECO:0000313|EMBL:ABP71740.1} OS=Rhodobacter sphaeroides (strain ATCC 17025 / ATH 2.4.3) GN=Rsph17025_2854 {ECO:0000313|EMBL:ABP71740.1} PE=4 SV=1

>tr|Q3IYX1|Q3IYX1_RHOS4 Formate dehydrogenase beta subunit {ECO:0000313|EMBL:ABA80263.1} OS=Rhodobacter sphaeroides (strain ATCC 17023 / 2.4.1 / NCIB 8253 / DSM 158) GN=fdsB {ECO:0000313|EMBL:ABA80263.1} PE=4 SV=1

>tr|A0A1S6IIY5|A0A1S6IIY5_9RHOB Formate dehydrogenase {ECO:0000313|EMBL:AQS49380.1} OS=Thioclava nitratireducens GN=BMG03_17470 {ECO:0000313|EMBL:AQS49380.1} PE=4 SV=1

>tr|A0A0K0Y4I2|A0A0K0Y4I2_9RHOB NADP-reducing hydrogenase subunit HndC {ECO:0000313|EMBL:AKS45771.1} OS=Octadecabacter temperatus GN=hndC {ECO:0000313|EMBL:AKS45771.1} PE=4 SV=1

>tr|A0A0B5DYL4|A0A0B5DYL4_9RHOB NADH dehydrogenase (Quinone) {ECO:0000313|EMBL:AJE46260.1} OS=Celeribacter indicus GN=P73_1545 {ECO:0000313|EMBL:AJE46260.1} PE=4 SV=1

>tr|A0A024EFU8|A0A024EFU8_9PSED Formate dehydrogenase, beta subunit {ECO:0000313|EMBL:AHZ71672.1} OS=Pseudomonas mandelii JR-1 GN=OU5_4593 {ECO:0000313|EMBL:AHZ71672.1} PE=4 SV=1

>tr|A0A0F7Y0W3|A0A0F7Y0W3_9PSED Formate dehydrogenase subunit beta {ECO:0000313|EMBL:CRI58021.1} OS=Pseudomonas sp. CCOS 191 GN=CCOS191_3485 {ECO:0000313|EMBL:CRI58021.1} PE=4 SV=1

>tr|Q2GAE3|Q2GAE3_NOVAD Formate dehydrogenase beta subunit {ECO:0000313|EMBL:ABD25180.1} OS=Novosphingobium aromaticivorans (strain ATCC 700278 / DSM 12444 / CIP 105152 / NBRC 16084 / F199) GN=Saro_0733 {ECO:0000313|EMBL:ABD25180.1} PE=4 SV=1

>tr|A0A1C7D4Q0|A0A1C7D4Q0_9SPHN NADP-reducing hydrogenase subunit HndC {ECO:0000313|EMBL:ANU06429.1} OS=Altererythrobacter namhicola GN=hndC {ECO:0000313|EMBL:ANU06429.1} PE=4 SV=1

>tr|E4PMM9|E4PMM9_MARAH NADH dehydrogenase (Quinone) {ECO:0000313|EMBL:ADP99937.1} OS=Marinobacter adhaerens (strain DSM 23420 / HP15) GN=HP15_4173 {ECO:0000313|EMBL:ADP99937.1} PE=4 SV=1

>tr|A0A0G3XHZ5|A0A0G3XHZ5_9SPHN Formate dehydrogenase {ECO:0000313|EMBL:AKM10219.1} OS=Croceicoccus naphthovorans GN=AB433_09965 {ECO:0000313|EMBL:AKM10219.1} PE=4 SV=1

>tr|A0A0H4VX56|A0A0H4VX56_9SPHN NAD-dependent formate dehydrogenase beta subunit {ECO:0000313|EMBL:AKQ41668.1} OS=Erythrobacter atlanticus GN=CP97_05975 {ECO:0000313|EMBL:AKQ41668.1} PE=4 SV=1

>tr|A0A0M3T9K8|A0A0M3T9K8_9SPHN NAD-dependent formate dehydrogenase beta subunit {ECO:0000313|EMBL:ALE15472.1} OS=Altererythrobacter epoxidivorans GN=AMC99_00156 {ECO:0000313|EMBL:ALE15472.1} PE=4 SV=1

>tr|A0A159YZN7|A0A159YZN7_9RHOB NAD dependent formate dehydrogenase, beta subunit {ECO:0000313|EMBL:AMY68082.1} OS=Defluviimonas alba GN=AKL17_0823 {ECO:0000313|EMBL:AMY68082.1} PE=4 SV=1

>tr|A0A1D9M944|A0A1D9M944_9RHOB Formate dehydrogenase {ECO:0000313|EMBL:AOZ68357.1} OS=Rhodobacter sp. LPB0142 GN=LPB142_02700 {ECO:0000313|EMBL:AOZ68357.1} PE=4 SV=1

>tr|A0A0P0ACN2|A0A0P0ACN2_9RHOB NAD-dependent formate dehydrogenase beta subunit {ECO:0000313|EMBL:ALI56692.1} OS=Celeribacter marinus GN=IMCC12053_2745 {ECO:0000313|EMBL:ALI56692.1} PE=4 SV=1

>tr|A0A2H5EZR4|A0A2H5EZR4_9RHOB Formate dehydrogenase {ECO:0000313|EMBL:AUH64789.1} OS=Paracoccus zhejiangensis GN=CX676_11935 {ECO:0000313|EMBL:AUH64789.1} PE=4 SV=1

>tr|A0A1V0RUY6|A0A1V0RUY6_9RHOB NADP-reducing hydrogenase subunit HndC {ECO:0000313|EMBL:ARE85392.1} OS=Roseovarius mucosus GN=hndC {ECO:0000313|EMBL:ARE85392.1} PE=4 SV=1

>tr|A0A1S6IC74|A0A1S6IC74_9RHOB Formate dehydrogenase {ECO:0000313|EMBL:AQS47089.1} OS=Thioclava nitratireducens GN=BMG03_04220 {ECO:0000313|EMBL:AQS47089.1} PE=4 SV=1

>tr|A0A1V0GQ36|A0A1V0GQ36_9RHOB Formate dehydrogenase {ECO:0000313|EMBL:ARC35799.1} OS=Paracoccus yeei GN=A6J80_04830 {ECO:0000313|EMBL:ARC35799.1} PE=4 SV=1

>tr|A0A291GF32|A0A291GF32_9RHOB Formate dehydrogenase {ECO:0000313|EMBL:ATG49153.1} OS=Celeribacter ethanolicus GN=CEW89_17210 {ECO:0000313|EMBL:ATG49153.1} PE=4 SV=1

>tr|M9R3W3|M9R3W3_9RHOB NADH-quinone oxidoreductase subunit F {ECO:0000313|EMBL:AGI67339.1} OS=Octadecabacter antarcticus 307 GN=nuoF1 {ECO:0000313|EMBL:AGI67339.1} PE=4 SV=1

>tr|A0A1W6CYI1|A0A1W6CYI1_9RHOB Formate dehydrogenase {ECO:0000313|EMBL:ARJ69859.1} OS=Paracoccus contaminans GN=B0A89_09710 {ECO:0000313|EMBL:ARJ69859.1} PE=4 SV=1

>tr|A0A1V0Q0G6|A0A1V0Q0G6_9RHOB NAD-dependent formate dehydrogenase beta subunit {ECO:0000313|EMBL:ARE41648.1} OS=Rhodovulum sp. P5 GN=RGUI_3507 {ECO:0000313|EMBL:ARE41648.1} PE=4 SV=1

>tr|A0A1V0HWF0|A0A1V0HWF0_9RHOB Formate dehydrogenase {ECO:0000313|EMBL:ARC88286.1} OS=Rhodovulum sp. MB263 GN=B5V46_06510 {ECO:0000313|EMBL:ARC88286.1} PE=4 SV=1

>tr|A0A0D6B107|A0A0D6B107_RHOSU NADH dehydrogenase {ECO:0000313|EMBL:BAQ68570.1} OS=Rhodovulum sulfidophilum GN=NHU_01411 {ECO:0000313|EMBL:BAQ68570.1} PE=4 SV=1

>tr|W8S954|W8S954_9RHOB NAD-dependent formate dehydrogenase beta subunit {ECO:0000313|EMBL:AHM05491.1} OS=Roseibacterium elongatum DSM 19469 GN=roselon_03228 {ECO:0000313|EMBL:AHM05491.1} PE=4 SV=1

>tr|Q28P86|Q28P86_JANSC Formate dehydrogenase beta subunit {ECO:0000313|EMBL:ABD55476.1} OS=Jannaschia sp. (strain CCS1) GN=Jann_2559 {ECO:0000313|EMBL:ABD55476.1} PE=4 SV=1

>tr|D5AQH1|D5AQH1_RHOCB NAD-dependent formate dehydrogenase, beta subunit {ECO:0000313|EMBL:ADE86760.1} OS=Rhodobacter capsulatus (strain ATCC BAA-309 / NBRC 16581 / SB1003) GN=fdhB {ECO:0000313|EMBL:ADE86760.1} PE=4 SV=1

>tr|F3LUW7|F3LUW7_9BURK NADH dehydrogenase (Quinone) {ECO:0000313|EMBL:EGJ12135.1} OS=Rubrivivax benzoatilyticus JA2 = ATCC BAA-35 GN=RBXJA2T_17474 {ECO:0000313|EMBL:EGJ12135.1} PE=4 SV=1

>tr|I0HVG7|I0HVG7_RUBGI NAD-dependent formate dehydrogenase, beta subunit FdsB {ECO:0000313|EMBL:BAL97004.1} OS=Rubrivivax gelatinosus (strain NBRC 100245 / IL144) GN=fdsB {ECO:0000313|EMBL:BAL97004.1} PE=4 SV=1

>tr|D8PEJ8|D8PEJ8_9BACT Formate dehydrogenase, beta subunit {ECO:0000313|EMBL:CBK41657.1} OS=Nitrospira defluvii GN=fdsB {ECO:0000313|EMBL:CBK41657.1} PE=4 SV=1

Supplementary data 1.4: Gene calling of *Methanosarcina acetivorans str. C2A*. Picture A shows the GenBank file from NCBI. At two places an amber stopcodon is present within the genes. B show the GenBank file from Metascan, where this stopcodon is actually used a stopcodon. Below, the incorporation of either amino acid X or O (pyrrolysine) in GenBank.

A

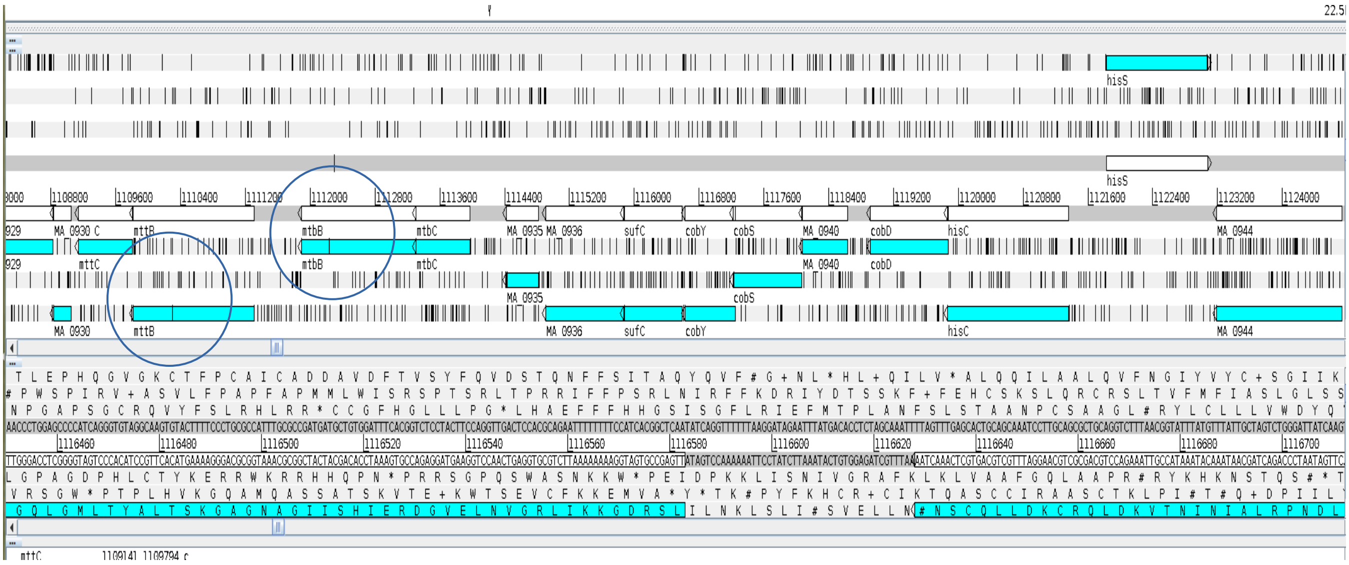

B

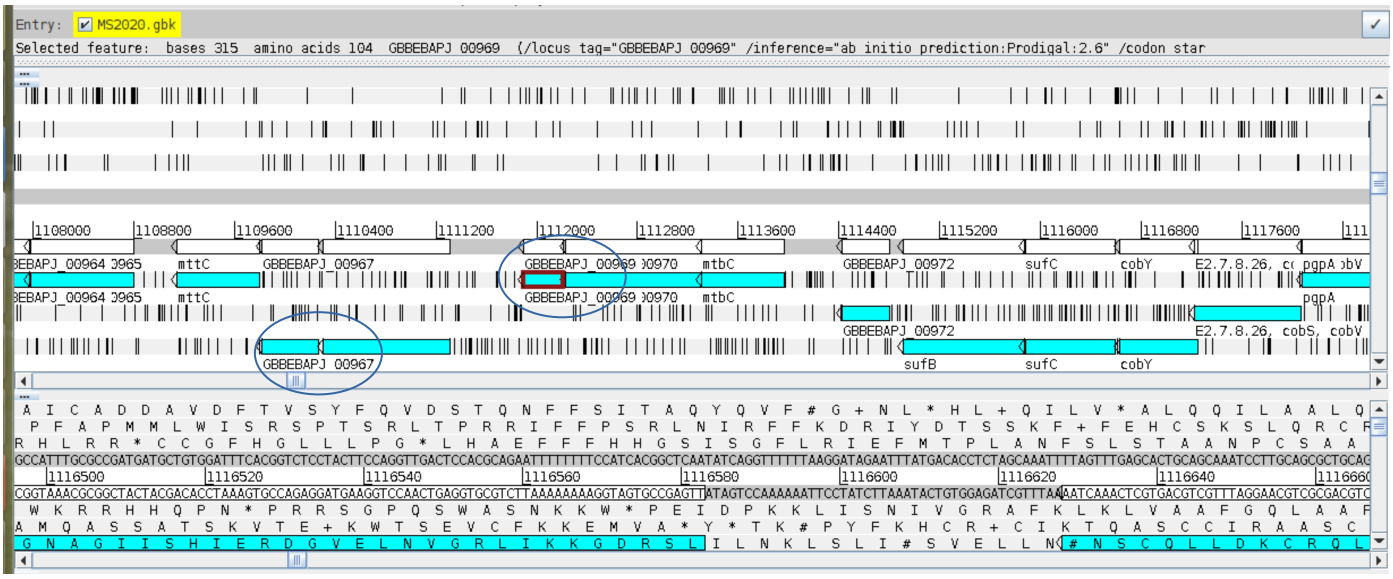


```
Sequence ID: AAM04365.1   Length: 495   Number of Matches: 1

Range 1: 1 to 495  GenPept  Graphics                 ▼ Next Match  ▲ Previous

Score            Expect  Method                           Identities     Positives      Gaps
1022 bits(2643)  0.0     Compositional matrix adjust.     494/495(99%)   494/495(99%)   1/495(0%)

Query  1    MAQNNAVAGFSSLQGVELNLFTIDELKAIHYATMEVLMNPGVQVSDPEARQIFKENGCEV  60
            MAQNNAVAGFSSLQGVELNLFTIDELKAIHYATMEVLMNPGVQVSDPEARQIFKENGCEV
Sbjct  1    MAQNNAVAGFSSLQGVELNLFTIDELKAIHYATMEVLMNPGVQVSDPEARQIFKENGCEV  60

Query  61   DEKTNVVKIPEYLVRRALQLAPSRFVLWGRDKKYNTVQEAGGKVHWTCFGTGVKMCKYQS  120
            DEKTNVVKIPEYLVRRALQLAPSRFVLWGRDKKYNTVQEAGGKVHWTCFGTGVKMCKYQS
Sbjct  61   DEKTNVVKIPEYLVRRALQLAPSRFVLWGRDKKYNTVQEAGGKVHWTCFGTGVKMCKYQS  120

Query  121  GKYVTVDSVEQDIADIAKLCDWTENIDYFSLPVSARDWAGKGAQDVHETLTPIANTAKHY  180
            GKYVTVDSVEQDIADIAKLCDWTENIDYFSLPVSARDWAGKGAQDVHETLTPIANTAKHY
Sbjct  121  GKYVTVDSVEQDIADIAKLCDWTENIDYFSLPVSARDWAGKGAQDVHETLTPIANTAKHY  180

Query  181  HHIDPVGENVEYYRDIVKAYYGGDEEEARKKPIFSMLLCPTSPLELSVNACQVIIKGARF  240
            HHIDPVGENVEYYRDIVKAYYGGDEEEARKKPIFSMLLCPTSPLELSVNACQVIIKGARF
Sbjct  181  HHIDPVGENVEYYRDIVKAYYGGDEEEARKKPIFSMLLCPTSPLELSVNACQVIIKGARF  240

Query  241  GMPVNVLSMAMSGGSSPVYLAGTLVTHNAEVLSGIVLAQLTVPGAKVWYGSSTTTFDLKK  300
            GMPVNVLSMAMSGGSSPVYLAGTLVTHNAEVLSGIVLAQLTVPGAKVWYGSSTTTFDLKK
Sbjct  241  GMPVNVLSMAMSGGSSPVYLAGTLVTHNAEVLSGIVLAQLTVPGAKVWYGSSTTTFDLKK  300

Query  301  GTAPVGSPELGLISAAVAKLAQFYGLPSYVAGT-ADAKIPDNQTGHEKTMTCFLPALAGA  359
            GTAPVGSPELGLISAAVAKLAQFYGLPSYVAGT ADAKIPDNQTGHEKTMTCFLPALAGA
Sbjct  301  GTAPVGSPELGLISAAVAKLAQFYGLPSYVAGTXADAKIPDNQTGHEKTMTCFLPALAGA  360

Query  360  NTIYGAGMLELGMTFSMEQLVIDNDIIKMVKKAMQGIEVSPETLAVDSIQKVGIGNNFLA  419
            NTIYGAGMLELGMTFSMEQLVIDNDIIKMVKKAMQGIEVSPETLAVDSIQKVGIGNNFLA
Sbjct  361  NTIYGAGMLELGMTFSMEQLVIDNDIIKMVKKAMQGIEVSPETLAVDSIQKVGIGNNFLA  420

Query  420  LKQTRLLVNYPSDPMLIDRRMYGDWAASGSKDLAAVANEKVTDVLKHHEVPPIDTDILKD  479
            LKQTRLLVNYPSDPMLIDRRMYGDWAASGSKDLAAVANEKVTDVLKHHEVPPIDTDILKD
Sbjct  421  LKQTRLLVNYPSDPMLIDRRMYGDWAASGSKDLAAVANEKVTDVLKHHEVPPIDTDILKD  480

Query  480  MQAIVDRADKAFKES  494
            MQAIVDRADKAFKES
Sbjct  481  MQAIVDRADKAFKES  495
```

GBBEBAPJ_0096-7/8

Trimethylamine:corrinoid methyltransferase [Methanosarcina mazei SarPi]

Sequence ID: AKB62494.1 Length: 495 Number of Matches: 1

Range 1: 1 to 494 GenPept Graphics ▼ Next Match ▲ Previous

Score	Expect	Method	Identities	Positives	Gaps
972 bits(2514)	0.0	Compositional matrix adjust.	464/494(94%)	482/494(97%)	1/494(0%)

```
Query  1    MAQNNAVAGFSSLQGVELNLFTIDELKAIHYATMEVLMNPGVQVSDPEARQIFKENGCEV  60
            MAQNNAVAGF++L GVEL+LFT DELKAIHYATMEVLMNPGVQVSDPEARQIFKENGCEV
Sbjct  1    MAQNNAVAGFNALNGVELSLFTTDELKAIHYATMEVLMNPGVQVSDPEARQIFKENGCEV  60

Query  61   DEKTNVVKIPEYLVRRALQLAPSRFVLWGRDKKYNTVQEAGGKVHWTCFGTGVKMCKYQS  120
            DEKT++VKIPEYLVRRALQLAPSRFVLWGRDKKYNTVQEAGGKVHWTCFGTGVKMCKYQ
Sbjct  61   DEKTSIVKIPEYLVRRALQLAPSRFVLWGRDKKYNTVQEAGGKVHWTCFGTGVKMCKYQD  120

Query  121  GKYVTVDSVEQDIADIAKLCDWTENIDYFSLPVSARDWAGKGAQDVHETLTPIANTAKHY  180
            GKYVTVDSVEQDIADIAKLCDW ENIDYFSLPVSARDWAGKGAQDVHETLTPIANTAKHY
Sbjct  121  GKYVTVDSVEQDIADIAKLCDWAENIDYFSLPVSARDWAGKGAQDVHETLTPIANTAKHY  180

Query  181  HHIDPVGENVEYYRDIVKAYYGGDEEEARKKPIFSMLLCPTSPLELSVNACQVIIKGARF  240
            HHIDPVGE V+YYRDIVKAYYGGDEEEARKKPIFSMLLCPTSPLELSVNACQVII+GARF
Sbjct  181  HHIDPVGEQVDYYRDIVKAYYGGDEEEARKKPIFSMLLCPTSPLELSVNACQVIIRGARF  240

Query  241  GMPVNVLSMAMSGGSSPVYLAGTLVTHNAEVLSGIVLAQLTVPGAKVWYGSSTTTFDLKK  300
            GMPVNVLSMAMSGGSSPVYLAGTLVTHNAEVLSGIVLAQLTVPGAKVWYGSSTTTFDLKK
Sbjct  241  GMPVNVLSMAMSGGSSPVYLAGTLVTHNAEVLSGIVLAQLTVPGAKVWYGSSTTTFDLKK  300

Query  301  GTAPVGSPELGLISAAVAKLAQFYGLPSYVAGT-ADAKIPDNQTGHEKTMTCFLPALAGA  359
            GTAPVGSPELGLISAAVAKLAQFYGLPSYVAGT +DAKIPDNQ GHEKTMTC LPALAGA
Sbjct  301  GTAPVGSPELGLISAAVAKLAQFYGLPSYVAGTOSDAKIPDNQAGHEKTMTCLLPALAGA  360

Query  360  NTIYGAGMLELGMTFSMEQLVIDNDIIKMVKKAMQGIEVSPETLAVDSIQKVGIGNNFLA  419
            NTIYGAGMLELGMTFSMEQLVIDNDIIKMVKKAMQGI VSPETLAV+SIQKVGIGNNFLA
Sbjct  361  NTIYGAGMLELGMTFSMEQLVIDNDIIKMVKKAMQGIPVSPETLAVESIQKVGIGNNFLA  420

Query  420  LKQTRLLVNYPSDPMLIDRRMYGDWAASGSKDLAAVANEKVTDVLKHHEVPPIDTDILKD  479
            LKQTR+LV+YPS PMLIDRRM+GDWAASGSKDLAAVANEKV D+LK+H+VPP+D DILKD
Sbjct  421  LKQTRMLVDYPSSPMLIDRRMFGDWAASGSKDLAAVANEKVQDILKNHQVPPVDADILKD  480

Query  480  MQAIVDRADKAFKE  493
            MQAIVD+AD+AFKE
Sbjct  481  MQAIVDKADRAFKE  494
```

GBBEBAPJ_009-69/70/71

Supplementary file 1.5: Cycles diagram Anantharaman metagenome

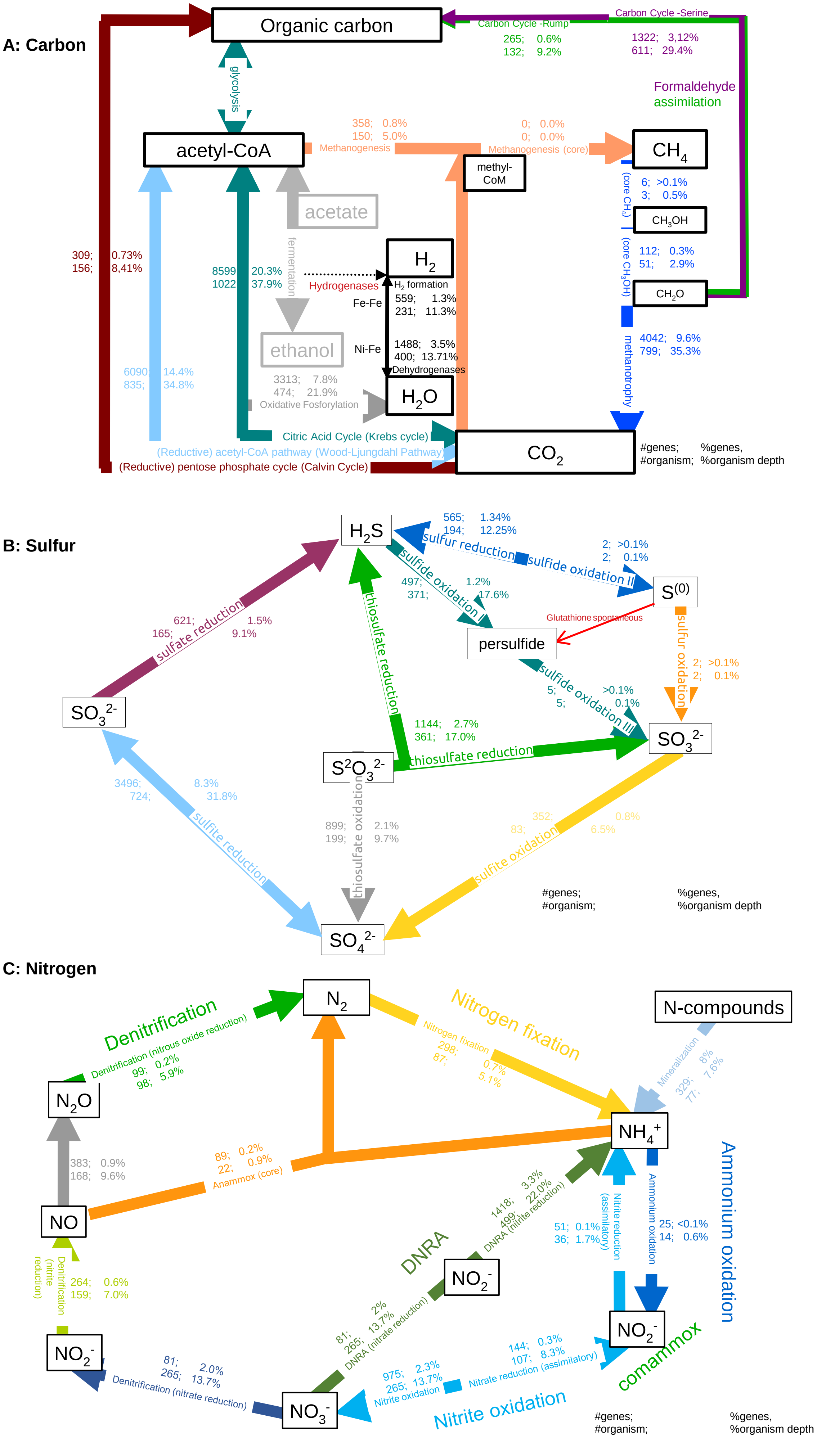

Supplementary file 1.6: PQQ-MDH-Anantharaman phylogenetic tree

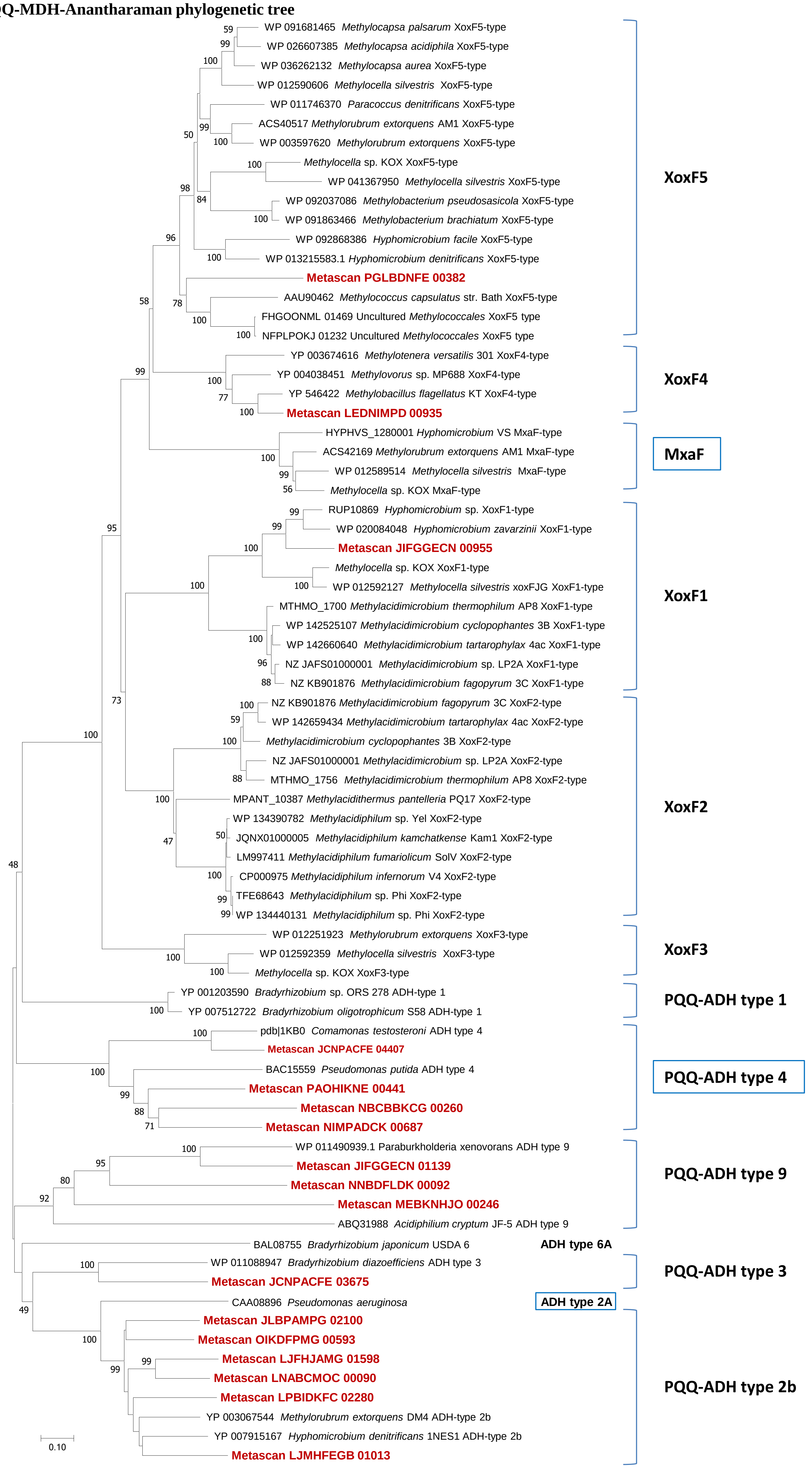

Supplementary file 1.6: Detailed phylogenetic analysis of MxaF-, XoxF and ADH-type PQQ-dependent alcohol dehydrogenase large subunit. Different branches of proteins that emerge from the tree are bracketed at the right side of the panel together with the taxonomic classification of their representatives; types that contain a catalytic calcium are framed. Metascan proteins from the Anantharaman dataset are in dark red bold. The remaining types show the lanthanide binding motif. The evolutionary history was inferred using the Neighbor-Joining method [1]. The optimal tree with the sum of branch length = 18.42487845 is shown. The percentage of replicate trees in which the associated taxa clustered together in the bootstrap test (500 replicates) are shown next to the branches for values > 45 [2]. The tree is drawn to scale, with branch lengths in the same units as those of the evolutionary distances used to infer the phylogenetic tree. The evolutionary distances were computed using the JTT matrix-based method [3] and are in the units of the number of amino acid substitutions per site. The analysis involved 75 amino acid sequences. All ambiguous positions were removed for each sequence pair. There were a total of 992 positions in the final dataset. Evolutionary analyses were conducted in MEGA7 [4].

1. Saitou N. and Nei M. (1987). The neighbor-joining method: A new method for reconstructing phylogenetic trees. Molecular Biology and Evolution 4:406-425.
2. Felsenstein J. (1985). Confidence limits on phylogenies: An approach using the bootstrap. Evolution 39:783-791.
3. Jones D.T., Taylor W.R., and Thornton J.M. (1992). The rapid generation of mutation data matrices from protein sequences. Computer Applications in the Biosciences 8: 275-282.
4. Kumar S., Stecher G., and Tamura K. (2016). MEGA7: Molecular Evolutionary Genetics Analysis version 7.0 for bigger datasets. Molecular Biology and Evolution 33:1870-1874.
